# Comparison of Survival Among Adults With Rectal Cancer Who Have Undergone Laparoscopic vs Open Surgery: A Meta-analysis

**DOI:** 10.1001/jamanetworkopen.2022.10861

**Published:** 2022-05-09

**Authors:** Meng Kong, Hongyuan Chen, Keshu Shan, Hongguang Sheng, Leping Li

**Affiliations:** 1 Department of Gastrointestinal Surgery, Shandong Provincial Hospital Affiliated to Shandong First Medical University, Jinan, Shandong, China; 2 Department of Gastrointestinal Surgery, Shandong Provincial Hospital, Cheeloo College of Medicine, Shandong University, Jinan, Shandong, China

## Abstract

**Importance:**

Two large randomized clinical trials (RCTs) found that laparoscopic surgery failed to yield noninferior pathologic outcomes compared with open surgery for patients with rectal cancer. The results raised concerns regarding the effectiveness of the laparoscopic approach for patients with rectal cancer.

**Objective:**

To compare the long-term oncologic outcomes of laparoscopic and open surgery for patients with rectal cancer.

**Data Sources:**

PubMed, Web of Science, Embase, and Cochrane Central Register of Controlled Trials were searched from database inception to August 13, 2021. Studies published in English were retrieved.

**Study Selection:**

The meta-analysis included RCTs that compared laparoscopic surgery with open surgery for patients with rectal cancer and reported the outcome of disease-free survival (DFS) or overall survival (OS). The following exclusion criteria were used: (1) non-RCTs, (2) studies without long-term survival outcomes of interest, and (3) studies that did not report Kaplan-Meier survival curves.

**Data Extraction and Synthesis:**

This meta-analysis was performed in line with the Preferred Reporting Items for Systematic Reviews and Meta-analyses reporting guideline for individual participant data development groups. Individual participant data on DFS and OS were extracted from the published Kaplan-Meier survival curves. One-stage and 2-stage meta-analyses were performed.

**Main Outcomes and Measures:**

Meta-analyses were conducted for DFS and OS. Hazard ratios (HRs) were used as effective measures.

**Results:**

Of 8471 records screened, 10 articles with 12 RCTs and 3709 participants were selected. The reconstructed survival curves for the combined population showed that the 5-year estimated DFS rates were 72.2% (95% CI, 69.4%-74.8%) for the laparoscopic group and 70.1% (95% CI, 67.0%-73.0%) for the open surgery group, and the 5-year estimated OS rates were 76.2% (95% CI, 73.8%-78.5%) for the laparoscopic group and 72.7% (95% CI, 69.8%-75.3%) for open surgery group. In 1-stage meta-analyses, DFS had a nonsignificant HR of 0.92 (95% CI, 0.80-1.06; *P* = .26), which suggested that DFS in the laparoscopic and open surgery groups was comparable; however, OS was significantly better in the laparoscopic group (HR, 0.85; 95% CI, 0.74-0.97; *P* = .02). The results were confirmed by 2-stage meta-analyses and were validated by sensitivity analysis with large RCTs.

**Conclusions and Relevance:**

A similar DFS but significantly better OS were found for patients who have undergone laparoscopic surgery compared with open surgery for rectal cancer. These findings address concerns regarding the effectiveness of laparoscopic surgery and support the routine use of laparoscopic surgery for patients with rectal cancer.

## Key Points

**Question:**

What are the long-term outcomes of laparoscopic surgery compared with open surgery for patients with rectal cancer?

**Findings:**

In this meta-analysis of 12 randomized clinical trials with 3709 participants with individual patient data, there was no difference between groups in disease-free survival. Patients who underwent laparoscopic surgery had better overall survival.

**Meaning:**

The findings of this study support the routine use of laparoscopic surgery for rectal cancer.

## Teaser

This individual participant data meta-analysis uses time-to-event data to compare the long-term oncologic outcomes after laparoscopic and open surgery for adult patients with rectal cancer.

## Introduction

Rectal cancer is currently one of the leading causes of cancer-related deaths worldwide.^1^ Although much progress has been achieved with neoadjuvant chemoradiotherapy, surgery is still the most important treatment for patients with rectal cancer. In the era of minimally invasive surgery, comparable long-term outcomes between laparoscopic and open surgery for rectal cancer have been demonstrated by several worldwide randomized clinical trials (RCTs)^2,3,4^ during the past 15 years.

However, 2 RCTs (ACOSOG Z6051^5^ and ALaCaRT [Australasian Laparoscopic Cancer of the Rectum]^6^), which were designed to compare pathologic outcomes between the 2 surgical approaches, demonstrated that laparoscopic surgery failed to yield a noninferior rate of “successful resection,” which was considered as a surrogate end point for long-term outcomes and included negative circumferential resection margin, complete or near-complete total mesorectal excision, and negative distal margin. However, comparable long-term survival outcomes, including disease-free survival (DFS) and overall survival (OS), between the 2 approaches were found in the follow-up of the 2 RCTs.^7,8^ The authors of the 2 RCTs ascribed the contradiction between the surrogate end point and corresponding long-term outcomes to insufficient statistical power for long-term outcomes. Therefore, it is necessary to perform a meta-analysis to obtain enough power to support or oppose laparoscopic surgery for patients with rectal cancer in terms of long-term outcomes.

Numerous meta-analyses^9,10,11,12,13^ have been conducted to compare laparoscopic surgery with open surgery in terms of long-term outcomes. However, the method of the pooled analysis was inappropriate in most of the previous meta-analyses. These survival meta-analyses combined dichotomous data, which could lead to misleading results and are not recommended by the Cochrane handbook.^14^ Conversely, time-to-event data are the most appropriate data for survival meta-analysis; furthermore, individual participant data (IPD) are believed to be particularly advantageous for analysis of time-to-event data.^15^ Therefore, we performed an IPD meta-analysis using time-to-event data and focused on the long-term survival outcomes after laparoscopic or open surgery for adult patients with rectal cancer.

## Methods

This meta-analysis was performed in line with the Preferred Reporting Items for Systematic Reviews and Meta-analyses (PRISMA) reporting guideline for IPD development groups,^16^ and the protocol was prospectively registered at PROSPERO (registration CRD42020206839). Because this meta-analysis was based on previously published studies, ethical approval by the institutional ethics committee and informed consent from patients were not required.

### Selection Criteria

We included RCTs that compared laparoscopic surgery with open surgery for adult patients with rectal cancer and reported the outcome of DFS or OS. Conference abstracts and unpublished data were considered if they reported Kaplan-Meier survival curves. When the results of a single trial were reported more than once, the reports with the longest survival outcomes were retained for inclusion. The following exclusion criteria were used: (1) non-RCTs, (2) studies without long-term survival outcomes of interest, and (3) studies that did not report Kaplan-Meier survival curves.

### Search Strategy

PubMed, Web of Science (including Conference Proceedings Citation Index–Science), Embase, and Cochrane Central Register of Controlled Trials were searched from database inception to August 13, 2021. Studies published in English were retrieved. The searches were performed based on the PICOS (population, intervention, comparison, outcomes, and study design) criteria. We did not include the outcomes in the search strategy to ensure that relevant articles were not missed. The following MeSH terms or keywords were used with the RCT filter recommended by Cochrane: *rectal cancer*, *rectal neoplasms*, *open*, *laparoscopy*, and *minimally invasive*. The details of the search strategy are shown in eTable 1 in the Supplement. Reference lists of included studies and relevant reviews were also hand searched. The registration numbers of the potentially eligible trials were traced back to the registration databases to find the latest versions of the trials.

### Assessment of Bias and the Overall Quality of Evidence

Two authors (M.K. and K.S.) independently assessed the risk of bias for each included RCT using the Cochrane risk-of-bias tool^17^ with Review Manager, version 5.3.5 (The Cochrane Collaboration). The quality of evidence for outcomes was assessed by the Grading of Recommendations Assessment, Development, and Evaluation (GRADE) approach.^18^

### Data Extraction

Two of the authors (M.K. and H.C.) independently extracted the following data from each study: general study information, study design, inclusion and exclusion criteria, participant characteristics, sample size in each group, location of tumors, surgical approaches, conversion rates, neoadjuvant therapy, and follow-up durations.

The IPD information of DFS and OS was extracted from the published Kaplan-Meier survival curves using Engauge Digitizer, version 12.1.^19^ The extracted survival information and the published risk table were used to reconstruct the survival curve for each included study using the method of Wei and Royston.^20^ The risk tables were also generated. The hazard ratios (HRs) and 95% CIs between the laparoscopic and open surgery groups were calculated using the Cox proportional hazards regression model. We compared the reconstructed curves, risk tables, estimated HRs, and estimated 95% CIs with those in the original publications. The extraction of information was repeated if there were apparent discrepancies.

### One-Stage Meta-analysis

The IPD information of time-to-event data from all the included RCTs was combined, and Kaplan-Meier curves of DFS and OS were separately generated for the whole included population. The Cox-based shared-frailty model with trial as a random effect was used in the 1-stage meta-analysis to generate the overall HRs and 95% CIs between laparoscopic and open surgery.^21^ Heterogeneity across trials was assessed using the estimated between-study variance in random effects.^22^

### Two-Stage Meta-analysis

The estimated HRs and 95% CIs of each of the included studies were pooled using a fixed-effects model or a random-effects model based on the degree of heterogeneity. *P* < .05 was required for the overall HRs to be statistically significant. Sensitivity analysis that used studies with a sample size equal to or greater than 100 in each group was performed. Heterogeneity was assessed using the *I*^2^ and χ^2^ measures. Moderate, substantial, and considerable heterogeneity was considered when *I*^2^ was greater than 30%, 50%, and 75%, respectively.^23^ Potential publication biases were analyzed using funnel plots and the Egger test. *P* < .10 for the Egger test suggested the existence of potential publication bias. We used Stata, version 16.0 (StataCorp LLC) and R software, version 4.0.3 (R Group for Statistical Computing) for data analysis. The detailed methods are described in the eAppendix 1 in the Supplement.

## Results

### Study Selection and Study Characteristics

**Figure 1.**
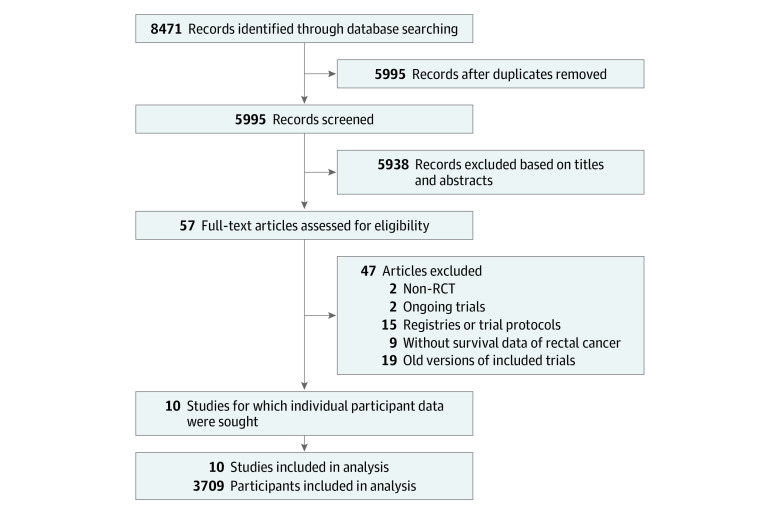
PRISMA Flow Diagram of Included Studies Ten articles with 12 randomized clinical trials (RCTs) and 3709 participants were selected.

After reviewing 8471 reports, 10 articles^2,3,4,7,8,24,25,26,27,28^ with 12 RCTs and 3709 participants were included in this meta-analysis (Figure 1). Ng et al^27^ reported the latest combined survival results of 3 RCTs, and the 3 RCTs were analyzed as 1 study in the following analyses because of the combined survival data and the same study design. The characteristics of the individual studies are presented in the Table,^2,3,4,7,8,24,25,26,27,28^ and the inclusion and exclusion criteria, as well as the follow-up schedule, are presented in eTable 2 in the Supplement. The 10 studies included 3709 participants, of whom 2097 were randomly assigned to laparoscopic surgery and 1612 were randomly assigned to open surgery. The included participants consisted of patients from Europe, North America, and East Asia. Both anterior resection and abdominoperineal resection were performed in all the included studies. All the trials had detailed follow-up schedules, and the median or mean follow-up durations varied from 32.8 to 143 months. For the outcomes, the CLASICC (Conventional Versus Laparoscopic-Assisted Surgery in Colorectal Cancer) trial^3^ reported DFS results but without survival curves. The ACOSOG Z6051 trial^7^ did not include OS as an outcome, and Liang et al^26^ reported OS but not DFS. In all, the IPD information was extracted from Kaplan-Meier curves for laparoscopic and open surgery groups in 8 studies for DFS and in 9 studies for OS.

**Table.** Characteristics of the Included Studies

Source	Country	Study interval	Study design	Approach	No.	Tumor location, No. (upper/mid/lower)	Surgical procedure, No. (AR/APR)	No./total No. (%)			Age, mean (SD), y	Male, No./total No. (%)	Follow-up, median (IQR), mo
								Conversion rate	Positive CRM	Neoadjuvant therapy			
Braga et al,^24^ 2007	Italy	2000-2003	Single center	Lap	83	30/NR/NR	76/7	6/83 (7.2)	NR	14/83 (16.9)	62.8 (12.6)	55/83 (66.3)	54.2 (NR)
				Open	85	24/NR/NR	74/11	NA	NR	12/85 (14.1)	65.3 (10.3)	64/85 (75.3)	
Lujan et al,^25^ 2009	Spain	2002-2007	Single center	Lap	97	0/NR/NR	77/24	8/101 (7.9)	4/101 (4.0)	73/101 (72.3)	67.8 (12.9)	62/101 (61.4)	32.8 (18.9)^a^
				Open	96	0/NR/NR	81/22	NA	3/103 (2.9)	77/104 (74.8)	66.0 (9.9)	64/103 (62.1)	34.1 (20.0)^a^
Liang et al,^26^ 2011	China	2004-2008	Single center	Lap	167	NR	86/83	1/169 (0.6)	NR	0/169	57.3 (14.1)^b^	104/169 (61.5)	44 (1-72)^c^
				Open	172	NR	104/70	NA	NR	0/174	57.3 (13.1)^b^	92/174 (52.9)	
Green et al,^3^ 2013	UK	1996-2002	Multicenter (27)	Lap	253	NR	167/63	82/242 (33.9)	30/193 (15.5)	NR	NR	NR	NR
				Open	128	NR	79/34	NA	14/97 (14.4)	NR	NR	NR	
Ng et al,^27^ 2014	China	1993-2007	Single center	Lap	136	60/36/40	96/40	21/136 (15.4)	2/136 (1.5)	0/136	63.9 (11.8)	74/136 (54.4)	101.6 (0.3-218.2)^c^
				Open	142	70/36/36	106/36	NA	2/142 (1.4)	0/142	64.9 (12.5)	87/142 (61.3)	106.5 (0.1-210.3)^c^
Kearney et al,^2^ 2015	8 countries^d^	2004-2010	Multicenter (30)	Lap	699	223/273/203	490/200	121/695 (17.4)	56/588 (9.5)	412/699 (58.9)	66.8 (10.5)	448/699 (64.1)	NR
				Open	344	116/136/93	265/80	NA	30/300 (10.0)	199/345 (57.7)	65.8 (10.9)	211/345 (61.2)	NR
Fleshman et al,^7^ 2019	US, Canada	2008-2013	Multicenter (35)	Lap	240	33/85/124	179/58	27/240 (11.3)	29/240 (12.1)	240/240 (100)	57.7 (11.5)	156/242 (64.5)	47.7 (26.1-59.1)
				Open	222	28/95/116	169/47	NA	17/222 (7.7)	222/222 (100)	57.2 (12.1)	158/239 (66.1)	48.1 (33.9-59.8)
Stevenson et al,^8^ 2019	Australia, New Zealand	2010-2014	Multicenter (24)	Lap	225	50/95/80	207/18	21/238 (8.8)	16/238 (6.7)	119/238 (49.8)	65 (56-74)^e^	160/238 (66.7)	38.4 (36.0-49.2)
				Open	225	48/99/78	210/15	NA	7/235 (3.0)	116/235 (48.0)	65 (56-73)^e^	151/235 (64.4)	39.6 (36.0-54.6)
Fujii et al,^28^ 2021	Japan	2008-2012	Single center	Lap	29	NR	25/4	NR	NR	0/29	NR	NR	NR
				Open	28	NR	26/2	NA	NR	0/28	NR	NR	NR
Park et al,^4^ 2021	Korean	2006-2009	Multicenter (3)	Lap	168	0/68/100	151/19	2/170 (1.2)	5/168 (3.0)	168/168 (100)	57.8 (11.1)	109/168 (64.9)	143 (122-156)
				Open	170	0/65/105	146/24	NA	7/170 (4.1)	170/170 (100)	59.1 (9.9)	110/170 (64.7)	

Abbreviations: APR, abdominoperineal resection; AR, anterior resection; CRM, circumferential resection margin; Lap, laparoscopy; NA, not applicable; NR, not reported.

^a^ Reported as mean (SD).

^b^ Reported as median (SD).

^c^ Reported as median (range).

^d^ Belgium, Canada, Denmark, Germany, the Netherlands, Spain, Korea, and Sweden.

^e^ Reported as median (IQR).

The risk of bias in the included studies is summarized in eFigure 1 in the Supplement, and the results of each study are shown in eFigure 2 in the Supplement. According to the Cochrane risk-of-bias tool, none of the included trials were classified as having a high risk of bias for objective outcomes, even though there were blinding biases in most of the included trials.

### Reconstructed Survival Curves

**Figure 2.**
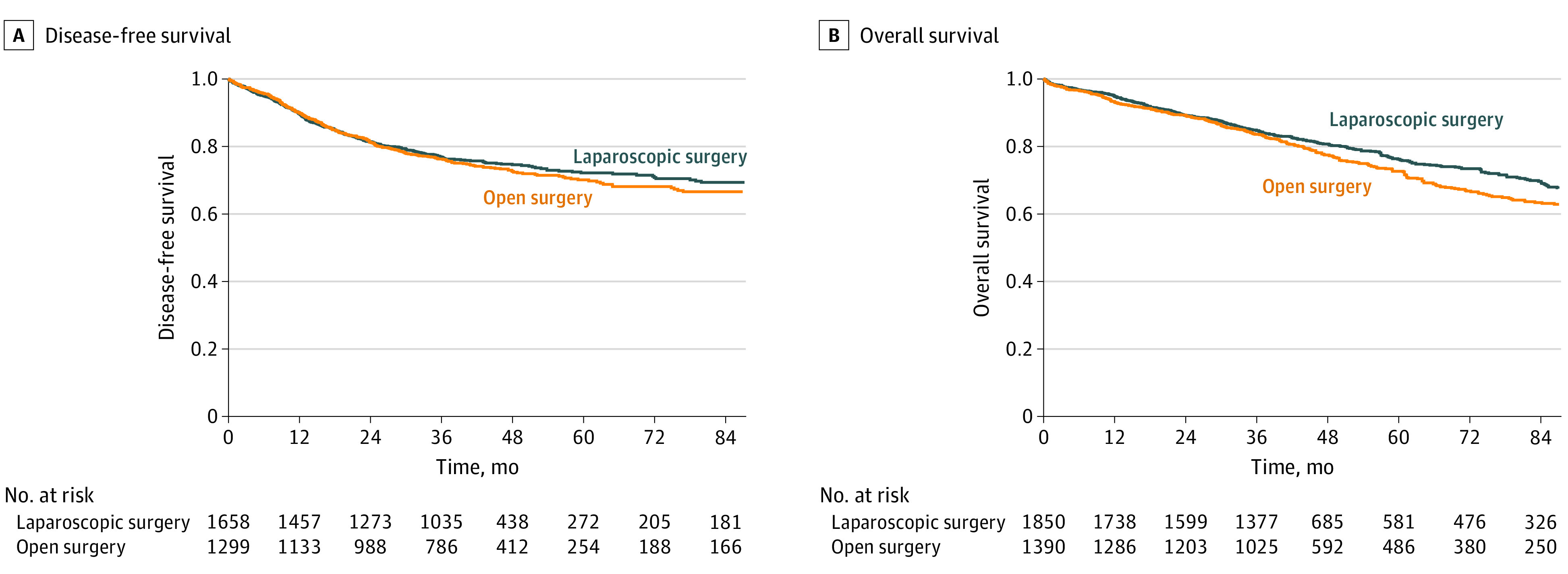
Reconstructed Kaplan-Meier Survival Curves and 1-Stage Meta-analysis A, The 5-year estimated disease-free survival (DFS) rates were 72.2% (95% CI, 69.4%-74.8%) for the laparoscopic group and 70.1% (95% CI, 67.0%-73.0%) for the open surgery group. One-stage meta-analysis of DFS yielded a hazard ratio (HR) of 0.92 (95% CI, 0.80-1.06; *P* = .26). B, The 5-year estimated overall survival (OS) rates were 76.2% (95% CI, 73.8%-78.5%) for the laparoscopic group and 72.7% (95% CI, 69.8%-75.3%) for the open surgery group. One-stage meta-analysis of OS yielded an HR of 0.85 (95% CI, 0.74-0.97; *P* = .02).

The reconstructed survival curve and side-by-side comparison with the original curve for each included study are presented in eAppendix 2 in the Supplement. The estimated and reported HRs are shown in eTable 3 in the Supplement. All the reconstructed Kaplan-Meier curves and the published curves in each of the studies were nearly identical, and the discrepancies in the risk tables were negligible. The reconstructed survival curves of DFS and OS for the combined population stratified by treatment groups are shown in Figure 2A and B. At 5 years, the estimated DFS rates were 72.2% (95% CI, 69.4%-74.8%) for the laparoscopic group and 70.1% (95% CI, 67.0%-73.0%) for the open surgery group. The difference between the 2 approaches in 5-year estimated OS was 3.5% (laparoscopic group, 76.2%; 95% CI, 73.8%-78.5%; open surgery group, 72.7%; 95% CI, 69.8%-75.3%).

### One-Stage Meta-analysis

In the Cox-based shared-frailty model, the meta-analysis of DFS yielded a nonsignificant HR of 0.92 (95% CI, 0.80-1.06; *P* = .26), which suggested that the DFS rates in the laparoscopic and open surgery groups were comparable (Figure 2A). Compared with open surgery, laparoscopic surgery was associated with significantly better OS with an HR of 0.85 (95% CI, 0.74-0.97; *P* = .02) (Figure 2B).

### Two-Stage Meta-analysis

**Figure 3.**
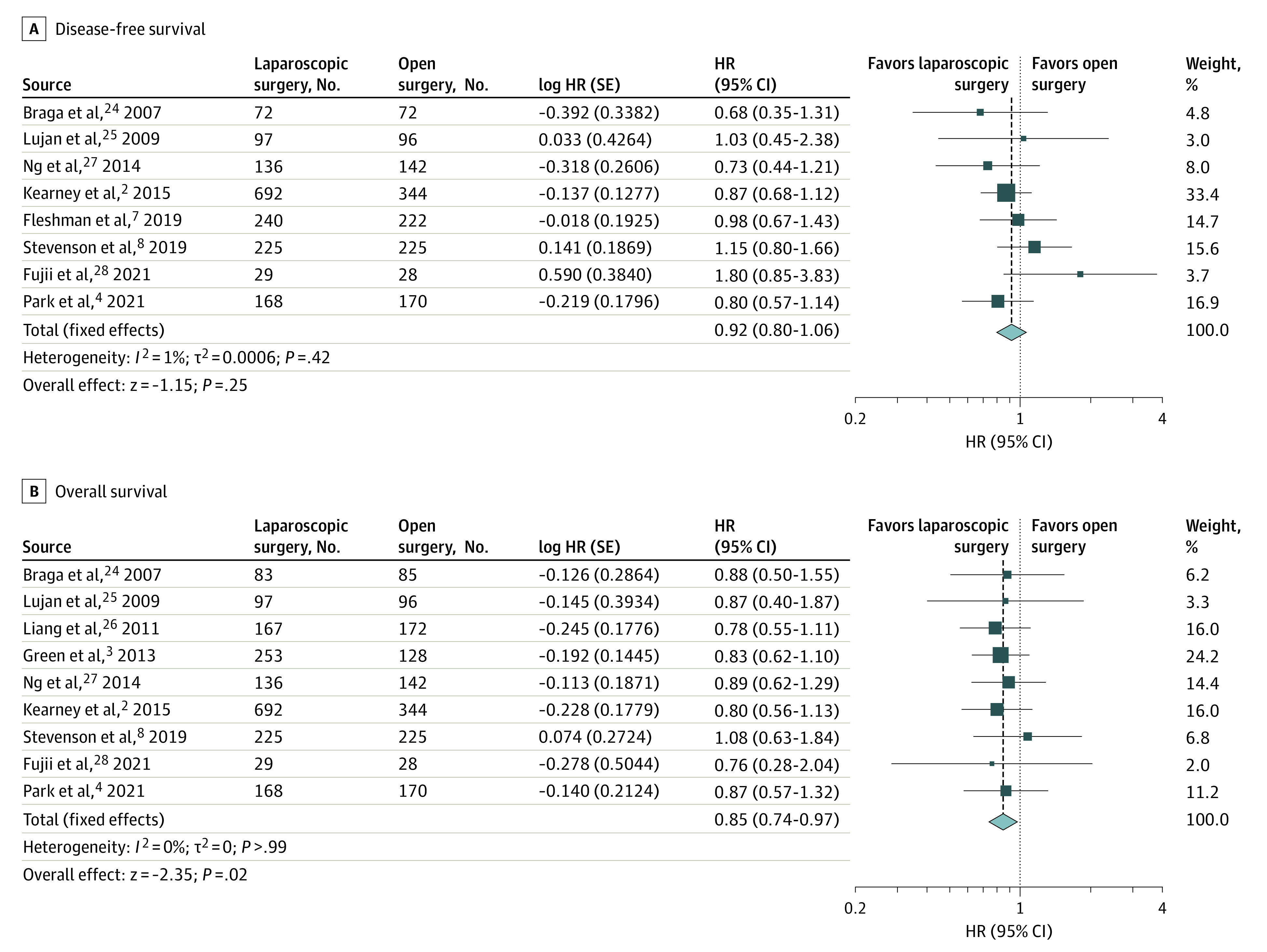
Two-Stage Meta-analysis A, Disease-free survival. B, Overall survival. A fixed-effects model with the inverse variance method was used for the meta-analysis. HR indicates hazard ratio.

To validate the robustness of the results, 2-stage meta-analyses were performed. For DFS, the pooled HR (0.92; 95% CI, 0.80-1.06; *P* = .25) was very similar to the HR of the 1-stage analysis (Figure 3A).^2,4,7,8,24,25,27,28^ The pooled HR of OS was 0.85 (95% CI, 0.74-0.97; *P* = .02), which was the same as the HR of OS in the 1-stage analysis (Figure 3B).^2,3,4,8,24,25,26,27,28^ Both the analyses of DFS and OS had low heterogeneities, at *I*^2^ = 1% and *I*^2^ = 0%, respectively.

Sensitivity analyses with large RCTs yielded similar pooled effect sizes (DFS: HR, 0.91; 95% CI, 0.78-1.06; *P* = .20; OS: HR, 0.84; 95% CI, 0.73-0.98; *P* = .03), with no heterogeneity (eFigure 3 in the Supplement). Furthermore, funnel plots and the Egger test revealed no publication bias for either the DFS or the OS outcome (eFigure 4 and eFigure 5 in the Supplement).

### Quality of Evidence for Outcomes

eTable 4 in the Supplement shows the assessment of the quality of evidence using the GRADE approach. Neither of the 2 outcomes was downgraded for risk of bias, inconsistency, indirectness, imprecision, or publication bias in the assessment. For both outcomes, the quality of evidence was judged to be high.

## Discussion

The present meta-analysis comparing laparoscopic vs open surgery for adult patients with rectal cancer included 12 RCTs with 3709 participants. The analysis showed that laparoscopic surgery was associated with a similar DFS but a significantly better OS than open surgery for adults with rectal cancer. To our knowledge, this was the first time that the association between laparoscopic rectal cancer resection and improved long-term outcome was shown using data from RCTs.

Three aspects of a new surgical approach for oncologic surgery need to be evaluated: short-term outcomes, pathologic outcomes, and long-term outcomes. Compared with traditional open surgery, favorable or noninferior short-term outcomes after laparoscopic surgery have been demonstrated in previous RCTs (CLASICC trial, COREAN [Comparison of Open Versus Laparoscopic Surgery for Mid and Low Rectal Cancer After Neoadjuvant Chemoradiotherapy] trial, and COLOR II [Colorectal Cancer Laparoscopic or Open Resection II] trial)^29,30,31^ and meta-analyses.^32,33^ These outcomes include blood loss, restoration of bowel function, length of hospital stay, complication rates, and postoperative mortality. Furthermore, these 3 large RCTs^29,30,31^ also found similar pathologic outcomes between the laparoscopic surgery and open surgery groups, such as a similar rate of positive circumferential resection margin and a similar rate of noncomplete mesorectal excision.

However, the comparable status of pathologic outcomes was not supported by the ACOSOG Z6051 trial^5^ or the ALaCaRT trial,^6^ both of which had noninferior designs and used a composite outcome of “successful resection” as a surrogate end point for survival outcomes. The inferior pathologic outcomes raised concern regarding the effectiveness of the laparoscopic approach for patients with rectal cancer. After the publication of the 2 RCTs, several meta-analyses were performed. A meta-analysis by Martínez-Pérez et al^34^ found a similar positive circumferential resection margin rate but a higher rate of noncomplete mesorectal excision in the laparoscopic surgery group than in the open surgery group. Another meta-analysis by Creavin et al^35^ performed subgroup analyses and showed that the higher rate of noncomplete mesorectal excision was attributed to superficial mesorectal defects but not deep mesorectal defects. They stated that the superficial defects may be caused by laparoscopic instruments and may not have negative effects on oncologic outcomes. Furthermore, a later meta-analysis by Acuna et al^32^ used a noninferiority approach and concluded that laparoscopy was noninferior to open surgery in terms of pathologic outcomes. In summary, even though 2 RCTs^5,6^ failed to show noninferior pathologic outcomes in the laparoscopic group, the results from meta-analyses tend to support the noninferior pathologic outcomes of laparoscopic surgery.

Consistent comparable long-term outcomes of laparoscopic and open surgery for rectal cancer have been reported across RCTs. A similar DFS rate was confirmed again in the present meta-analysis, and a significantly better OS was found with laparoscopic surgery than with open surgery for rectal cancer. The better OS may seem surprising, but we can find some clues in previous studies. Except for the ALaCaRT trial,^8^ all the included RCTs showed a trend favoring laparoscopic surgery in terms of OS (Figure 3B).^2,3,4,8,24,25,26,27,28^ For example, after 10 years of follow-up, the CLASICC trial^3^ reported median OS times of 82.7 months (IQR, 67.3-97.6) among the laparoscopic group and 65.8 months (IQR, 49.0-83.8) among the open surgery group. At 3 years after surgery, the COLOR II trial^2^ had OS rates of 86.7% in the laparoscopic group and 83.6% in the open surgery group. Furthermore, the COREAN trial^4^ reported a 5-year OS rate showing a 5.1% advantage of laparoscopic surgery compared with open surgery (87.5% vs 82.4%). However, none of the differences were statistically significant.

In contrast, a few population-based studies have revealed the beneficial effect of laparoscopic surgery or minimally invasive surgery on OS for patients with rectal cancer. A study including 16 378 patients with rectal cancer from a nationwide database in Germany found that 5-year OS was 82.6% for laparoscopic surgery and 76.6% for open surgery, with *P* < .001 in both univariable and multivariable Cox proportional hazards regression analyses.^36^ Another study analyzed 31 190 patients in the National Cancer Database (2010-2015) who underwent resection for locally advanced rectal cancer and found a significant difference in 5-year OS between patents who underwent minimally invasive resection and patients who underwent open resection (75.6% and 69.8%, respectively).^37^ The discrepancy between the results from RCTs and the results from extremely large population-based studies may be due to the insufficient sample size of RCTs.

In fact, none of the included RCTs calculated the sample size based on the difference in OS rates between the 2 approaches. Therefore, the sample size in a single study may not be enough to distinguish the difference in OS. However, the present meta-analysis aggregated the survival data from 9 studies (11 RCTs) and could generate enough statistical power to find a significant difference. For the meta-analysis of OS, we included 3240 participants (1850 who underwent laparoscopic surgery and 1390 who underwent open surgery), which was more than 3 times the number of participants in the COLOR II trial^2^ (the largest RCT evaluating laparoscopic surgery for rectal cancer). The number of participants in this meta-analysis provided the 3.5% difference in the 5-year estimated OS rate between the laparoscopic and open groups with statistical significance (*P* = .02).

Several reasons may explain the OS benefit associated with laparoscopic surgery. First, the improved recovery after laparoscopic surgery^31^ could allow patients to receive adjuvant therapy earlier. The delay in postoperative chemotherapy was demonstrated to be associated with worse OS among patients with colorectal cancer.^38^ In addition, the lower stress responses and higher levels of immune function among patients undergoing minimally invasive surgery^39^ may contribute to the long-term survival advantage of laparoscopic rectal surgery compared with open surgery. Further studies are necessary to explore the specific mechanisms underlying the positive effect of laparoscopic surgery on OS.

The populations included in the trials varied in terms of age and use of neoadjuvant therapy. For instance, the Eld Lap trial^28^ was designed to explore the efficiency of laparoscopic surgery for older patients (≥75 years) with colorectal cancer. Some trials^26,27,28^ did not include any participants who received neoadjuvant therapy because of the early study period or the use of different guidelines. The diversity of the populations included in this meta-analysis may make the results more representative. However, the diversity also limited our ability to conduct subgroup analysis.

### Strengths and Limitations

This meta-analysis has some strengths, including our use of the most appropriate method to analyze the latest data (ie, the use of IPD). We have included the most recent publications of RCTs with the latest survival data. The survival curves in the publications allowed us to extract the individual participant survival data for each trial. The meta-analysis of individual participant time-to-event data enabled us to generate more robust results than traditional aggregate data meta-analysis. In addition, the results were validated by 2-stage meta-analyses and by sensitivity analyses with large RCTs. Furthermore, the low heterogeneity (*I*^2^ = 1% for DFS and *I*^2^ = 0% for OS) between studies and the high quality of evidence assessed by the GRADE approach strengthened the robustness of the results.

This meta-analysis also has several limitations. The IPD extracted from Kaplan-Meier curves provided only patient-level survival data rather than data on other covariates, such as age, sex, body mass index, tumor location, and neoadjuvant therapy. Because the laparoscopic procedure for patients with mid-lower rectal cancer, high body mass index, or neoadjuvant chemoradiotherapy is relatively difficult, surgeons have more concerns about the oncologic outcome of laparoscopic surgery in this patient population. However, the current IPD did not allow us to conduct such subgroup analyses, which could also not be performed based on insufficient study-level relevant data. A meta-analysis of IPD with baseline clinical characteristics obtained from the authors of each study is needed. Furthermore, a small number of participants included in the ACOSOG Z6051 trial who underwent other types of minimally invasive surgical procedures (hand-assisted surgery and robotic surgery) may cause potential bias for the outcome of DFS. Finally, there may be language bias in this meta-analysis because only studies published in English were included.

## Conclusions

In the present IPD meta-analysis of high-quality RCTs, a similar DFS but an significantly better OS were found for laparoscopic surgery compared with open surgery for adults with rectal cancer. The survival benefit of laparoscopic surgery is encouraging and supports the routine use of laparoscopic surgery for adult patients with rectal cancer in the era of minimally invasive surgery.

## Notes

Supplement. **eTable 1.** Detailed Search Strategy **eTable 2.** Inclusion and Exclusion Criteria, and Follow-up Schedule of Included Studies **eTable 3.** Estimated and Reported Hazard Ratios **eTable 4.** GRADE Evaluation of the Quality of Evidence **eFigure 1.** Risk of Bias Graph: Reviews of Authors’ Judgments About Each Risk of Bias Item Are Presented as Percentages Across all Included Studies **eFigure 2.** Risk of Bias Summary: Reviews of Authors’ Judgments About Each Risk of Bias Item for Each Included Study **eFigure 3.** Forest Plot of Sensitivity Analysis With Large RCTs for DFS (A) and OS (B) **eFigure 4.** Funnel Plot and Egger’s Test of DFS **eFigure 5.** Funnel Plot and Egger’s Test of OS **eAppendix 1.** Supplementary Methods
eReferences.
**eAppendix 2.** Reconstructed Survival Curve and Side-by-Side Comparison With the Original Curve for Each Included Study
