# Supplementary material for: Comparison of Survival Among Adults With Rectal Cancer Who Have Undergone Laparoscopic vs Open Surgery: A Meta-analysis
Source: JAMA Netw Open. 2022 May 9;5(5):e2210861. doi: 10.1001/jamanetworkopen.2022.10861 (PMC9086842; doi:10.1001/jamanetworkopen.2022.10861)
Supplement: Supplement. — eTable 1. Detailed Search Strategy eTable 2. Inclusion and Exclusion Criteria, and Follow-up Schedule of Included Studies eTable 3. Estimated and Reported Hazard Ratios eTable 4. GRADE Evaluation of the Quality of Evidence eFigure 1. Risk of Bias Graph: Reviews of Authors’ Judgments About Each Risk of Bias Item Are Presented as Percentages Across all Included Studies eFigure 2. Risk of Bias Summary: Reviews of Authors’ Judgments About Each Risk of Bias Item for Each Included Study eFigure 3. Forest Plot of Sensitivity Analysis With Large RCTs for DFS (A) and OS (B) eFigure 4. Funnel Plot and Egger’s Test of DFS eFigure 5. Funnel Plot and Egger’s Test of OS eAppendix 1. Supplementary Methods eReferences. eAppendix 2. Reconstructed Survival Curve and Side-by-Side Comparison With the Original Curve for Each Included Study [file jamanetwopen-e2210861-s001.pdf]

Supplementary Online Content

Kong M, Chen H, Shan K, Sheng H, Li L. Comparison of survival among adults with rectal cancer who have undergone laparoscopic vs open surgery: a meta-analysis. *JAMA Netw Open.* 2022;5(5):e2210861. doi:10.1001/jamanetworkopen.2022.10861

eTable 1. Detailed Search Strategy
eTable 2. Inclusion and Exclusion Criteria, and Follow-up Schedule of Included Studies
eTable 3. Estimated and Reported Hazard Ratios
eTable 4. GRADE Evaluation of the Quality of Evidence
eFigure 1. Risk of Bias Graph: Reviews of Authors' Judgments About Each Risk of Bias Item Are Presented as Percentages Across all Included Studies
eFigure 2. Risk of Bias Summary: Reviews of Authors' Judgments About Each Risk of Bias Item for Each Included Study
eFigure 3. Forest Plot of Sensitivity Analysis With Large RCTs for DFS (A) and OS (B)
eFigure 4. Funnel Plot and Egger's Test of DFS
eFigure 5. Funnel Plot and Egger's Test of OS
eAppendix 1. Supplementary Methods
eReferences.
eAppendix 2. Reconstructed Survival Curve and Side-by-Side Comparison With the Original Curve for Each Included Study

This supplementary material has been provided by the authors to give readers additional information about their work.

© 2022 Kong M et al. *JAMA Network Open.*

eTable 1. Detailed Search Strategy

Database	PICOS	Search strategy
PubMed	P	rectal cancer OR rectal tumor OR rectal tumour OR rectal neoplasms[MeSH Terms]
	I, C	laparoscopy[MeSH Terms] OR surgical procedures, minimally invasive[MeSH Terms] OR laparoscop* OR minimally invasive surgery OR laparoscopic surgery
	S	randomized controlled trial [pt] OR controlled clinical trial [pt] OR randomized [tiab] OR placebo [tiab] OR drug therapy [sh] OR randomly [tiab] OR trial [tiab] OR groups [tiab]) NOT (animals [mh] NOT humans [mh]
EMBASE	P	(rectal AND canc*) OR (rectal AND tumor) OR 'rectum cancer'/exp
	I, C	'laparoscopy'/exp OR 'minimally invasive surgery'/exp OR laparoscop* OR (minimally AND invasive AND surgery)
	S	'crossover procedure':de OR 'double-blind procedure':de OR 'randomized controlled trial':de OR 'single-blind procedure':de OR random*:de,ab,ti OR factorial*:de,ab,ti OR crossover*:de,ab,ti OR ((cross NEXT/1 over*):de,ab,ti) OR placebo*:de,ab,ti OR ((doubl* NEAR/1 blind*):de,ab,ti) OR ((singl* NEAR/1 blind*):de,ab,ti) OR assign*:de,ab,ti OR allocat*:de,ab,ti OR volunteer*:de,ab,ti
Web of Science	P	TS= (rectal canc* OR rectal tumor OR rectal tumour OR rectal neoplasms)
	I, C	TS= (laparoscopy OR surgical procedures, minimally invasive OR laparoscop* OR minimally invasive surgery OR laparoscopic surgery)
	S	TS= (randomized controlled trial OR controlled clinical trial OR randomized OR placebo OR drug therapy OR randomly OR trial OR groups）
CENTRAL	P	MeSH descriptor: [Rectal Neoplasms] explode all trees OR rectal tumor* OR rectal canc*
	I, C	laparoscop* OR MeSH descriptor: [Laparoscopy] explode all trees OR minimally invasive surgery

PICOS: population, intervention, comparison, outcomes and study design; CENTRAL: Cochrane Central Register of Controlled Trials.

© 2022 Kong M et al. *JAMA Network Open.*

eTable 2. Inclusion and Exclusion Criteria, and Follow-up Schedule of Included Studies

Study, Year	Age	Location	T4* stage or CRM positive	M1* stage	Other important exclusion criteria	Follow-up schedule
Braga et al, 2007	18 years or older	Upper	T4b was excluded	Included for OS, Excluded for DFS	Emergency surgery	Every 6 months
Lujan et al, 2009	NR	Mid and low	Excluded	Not excluded	Emergency surgery, FAP	Every 3 months for the first 2 years, and every 6 months thereafter
Liang et al, 2011	NR	NR	Not excluded	Excluded	BMI >30 kg/m^2	Every 3 months for the first 2 years, and every 6 months thereafter
CLASICC, 2013	NR	NR	Not excluded	Not excluded	Synchronous adenocarcinomas	Every 3 months for the first year, 4 months the second year, and 6 months thereafter
Ng et al, 2014	NR	Upper, mid and low	T4b was excluded	No M1 stage	Tumor larger than 6 cm, synchronous colorectal tumors	Every 3 months for the first 2 years, and every 6 months thereafter
COLOR II, 2015	NR	Upper, mid and low	Excluded	Excluded	T1 tumor treated with local transanal excision, FAP, HNPCC	At least once a year for 5 years
ACOSOG Z6051, 2019	18 years or older	Upper, mid and low	Excluded	Excluded	BMI >34 kg/m^2	Every 3 months for the first 1 years, and every 6 months thereafter
ALaCaRT, 2019	18 years or older	Upper, mid and low	Excluded	Excluded	Concurrent or previous invasive pelvic malignant tumors	Every 3 months for the first 1 years, and every 6 months thereafter

© 2022 Kong M et al. *JAMA Network Open.*

Eld Lap, 2020	75 years or older	Upper, mid and low	T4b was excluded	Excluded	Malignancy in another organ; tumor larger than 8 cm	Stage 0 or I, once a year for 5 years;<br>Stage II or IIIA, every 6 months for the first 2 years, once a year from years 3 to 5;<br>Stage IIIB or IIIC, every 4 months for the first 2 years, every 6 months from years 3 to 5
COREAN, 2021	18–80 years	Mid and low	Not excluded	Excluded	Another primary malignance	Every 3 months for the first 2 years, every 6 months for the next 3 years, and every 6 months or 1 year thereafter

* American Joint Committee on Cancer (AJCC) TNM stage

CRM: circumferential resection margin; FAP: familial adenomatous polyposis; HNPCC: hereditary nonpolyposis colorectal cancer; BMI: body mass index.

© 2022 Kong M et al. *JAMA Network Open.*

eTable 3. Estimated and Reported Hazard Ratios

Endpoint	Study, year	HR (95% CI)	
		Reported	Estimated
DFS	Braga et al, 2007	NR	0.676 (0.348-1.312)
	Lujan et al, 2009	NR	1.034 (0.448-2.384)
	Ng et al, 2014	NR	0.728 (0.437-1.212)
	COLOR II, 2015	NR	0.872 (0.679-1.120)
	ACOSOG Z6051, 2019	0.95 (0.65-1.37)	0.982 (0.674-1.432)
	ALa CaRT, 2019	1.17 (0.81-1.68)	1.152 (0.798-1.661)
	Eld Lap, 2020	NR	1.804 (0.850-3.830)
	COREAN, 2021	0.806(0.571-1.149)	0.803(0.565-1.143)
OS	Braga et al, 2007	NR	0.882 (0.503-1.546)
	Lujan et al, 2009	NR	0.865 (0.400-1.870)
	Liang et al, 2011	NR	0.783 (0.552-1.108)
	CLASICC, 2013	NR	0.826 (0.622-1.96)
	Ng et al, 2014	NR	0.893 (0.619-1.289)
	COLOR II, 2015	NR	0.796 (0.562-1.129)
	ALa CaRT, 2019	1.08 (0.63-1.86)	1.077 (0.631-1.836)
	Eld Lap, 2020	NR	0.757 (0.282-2.036)
	COREAN, 2021	0.877(0.585-1.316)	0.869(0.573-1.318)

DFS, disease-free survival; OS, overall survival; HR, hazard ratio; CI, confidence interval; NR, not reported.

© 2022 Kong M et al. *JAMA Network Open.*

eTable 4. GRADE Evaluation of the Quality of Evidence

Certainty assessment							No. of patients		Effect		Certainty	Importance
**No. of studies**	**Study design**	**Risk of bias**	**Inconsistency**	**Indirectness**	**Imprecision**	**Other considerations**	**laparoscopic surgery**	**Open surgery**	**Relative (95% CI)**	**Absolute (95% CI)**		
**DFS**												
8	randomised trials	not serious	not serious	not serious	not serious	none	1659 participants	1299 participants	HR 0.920 (0.796 to 1.063) [disease-free survival]	20 more per 1,000 (from 15 fewer to 52 more)	⊕⊕⊕⊕ HIGH	CRITICAL
**OS**												
9	randomised trials	not serious	not serious	not serious	not serious	none	1850 participants	1390 participants	HR 0.846 (0.736 to 0.973) [overall survival]	19 more per 1,000 (from 3 more to 33 more)	⊕⊕⊕⊕ HIGH	CRITICAL

DFS, disease-free survival; OS, overall survival; HR, hazard ratio; CI, confidence interval.

© 2022 Kong M et al. *JAMA Network Open.*

eFigure 1. Risk of Bias Graph: Reviews of Authors' Judgments About Each Risk of Bias Item Are Presented as Percentages Across all Included Studies

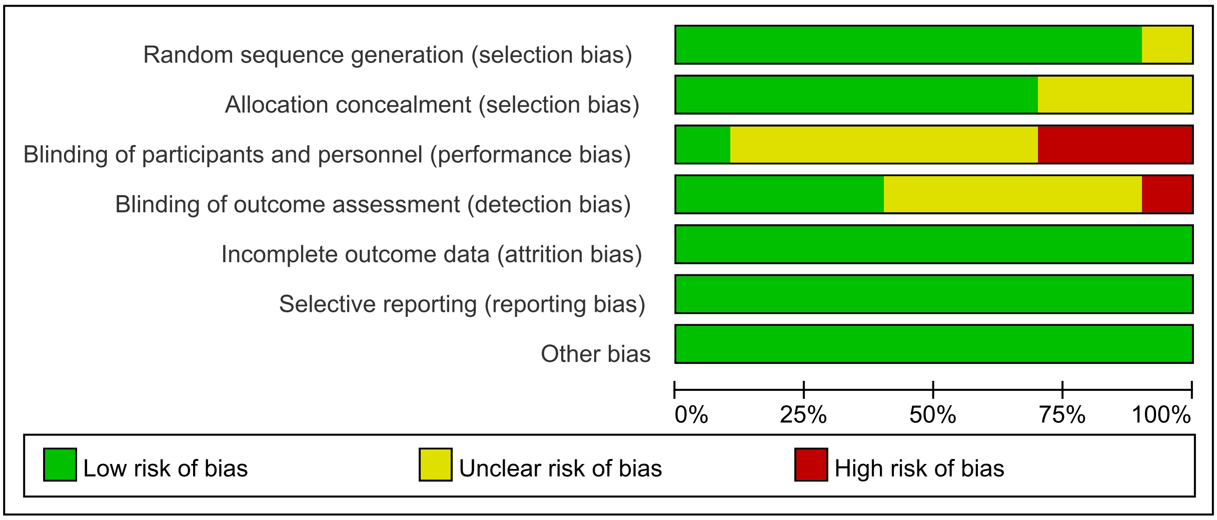

© 2022 Kong M et al. *JAMA Network Open.*

eFigure 2. Risk of Bias Summary: Reviews of Authors' Judgments About Each Risk of Bias Item for Each Included Study

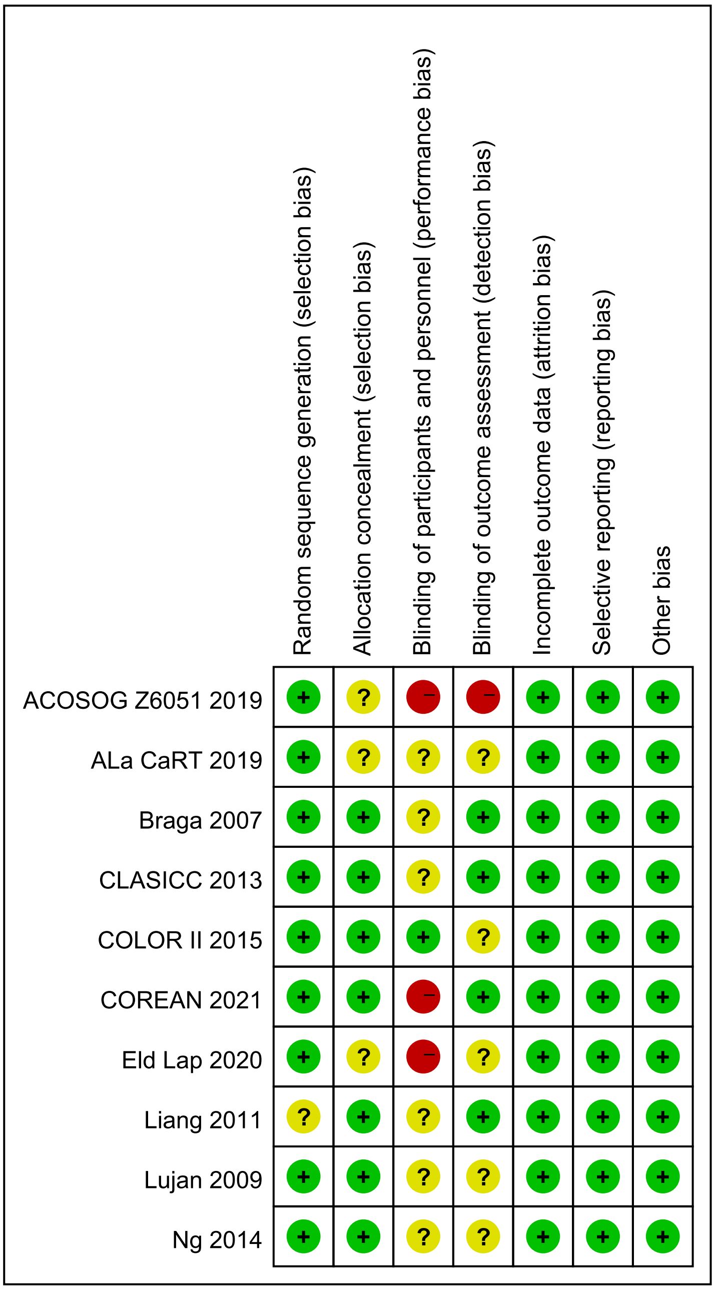

© 2022 Kong M et al. *JAMA Network Open.*

eFigure 3. Forest Plot of Sensitivity Analysis With Large RCTs for DFS (A) and OS (B)

A

Source	logHR(SE)	Lap, No.	Open, No.	HR(95%CI)	Weight,%
**Ng et al,[27] 2014**	−0.318 (0.2606)	136	142	0.73 (0.44−1.21)	9.1
**COLOR II,[2] 2015**	−0.137 (0.1277)	692	344	0.87 (0.68−1.12)	37.7
**ACOSOG Z6051,[7] 2019**	−0.018 (0.1925)	240	222	0.98 (0.67−1.43)	16.6
**ALaCaRT,[8] 2019**	0.141 (0.1869)	225	225	1.15 (0.80−1.66)	17.6
**COREAN,[4] 2021**	−0.219 (0.1796)	168	170	0.80 (0.57−1.14)	19.1
Total (fixed−effects)				0.90 (0.78−1.05)	100.0

Heterogeneity: I^2 = 0%, τ^2 = 0, p = .55
Test for overall effect: z = −1.28 (p = .20)

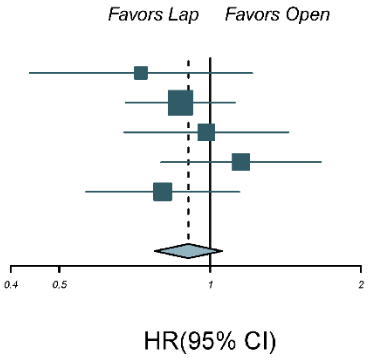

B

Source	logHR(SE)	Lap, No.	Open, No.	HR(95%CI)	Weight,%
**Liang et al,[26] 2011**	−0.245 (0.1776)	167	172	0.78 (0.55−1.11)	18.1
**CLASICC,[3] 2013**	−0.192 (0.1445)	253	128	0.83 (0.62−1.10)	27.3
**Ng et al,[27] 2014**	−0.113 (0.1871)	136	142	0.89 (0.62−1.29)	16.3
**COLOR II,[2] 2015**	−0.228 (0.1779)	692	344	0.80 (0.56−1.13)	18.0
**ALaCaRT,[8] 2019**	0.074 (0.2724)	225	225	1.08 (0.63−1.84)	7.7
**COREAN,[4] 2021**	−0.140 (0.2124)	168	170	0.87 (0.57−1.32)	12.6
Total(fixed−effects)				0.85 (0.73−0.98)	100.0

Heterogeneity: I^2 = 0%, τ^2 = 0, p = .94
Test for overall effect: z = −2.23 (p = .03)

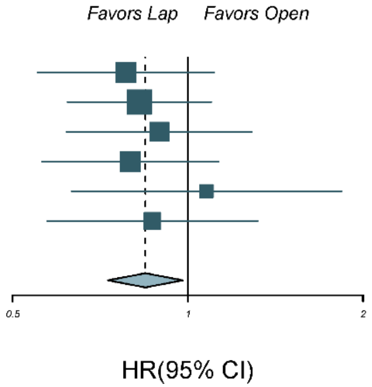

Abbreviations: DFS, disease-free survival; OS, overall survival; Lap, laparoscopy; HR, hazard ratio; CI, confidence interval.

© 2022 Kong M et al. *JAMA Network Open.*

eFigure 4. Funnel Plot and Egger's Test of DFS

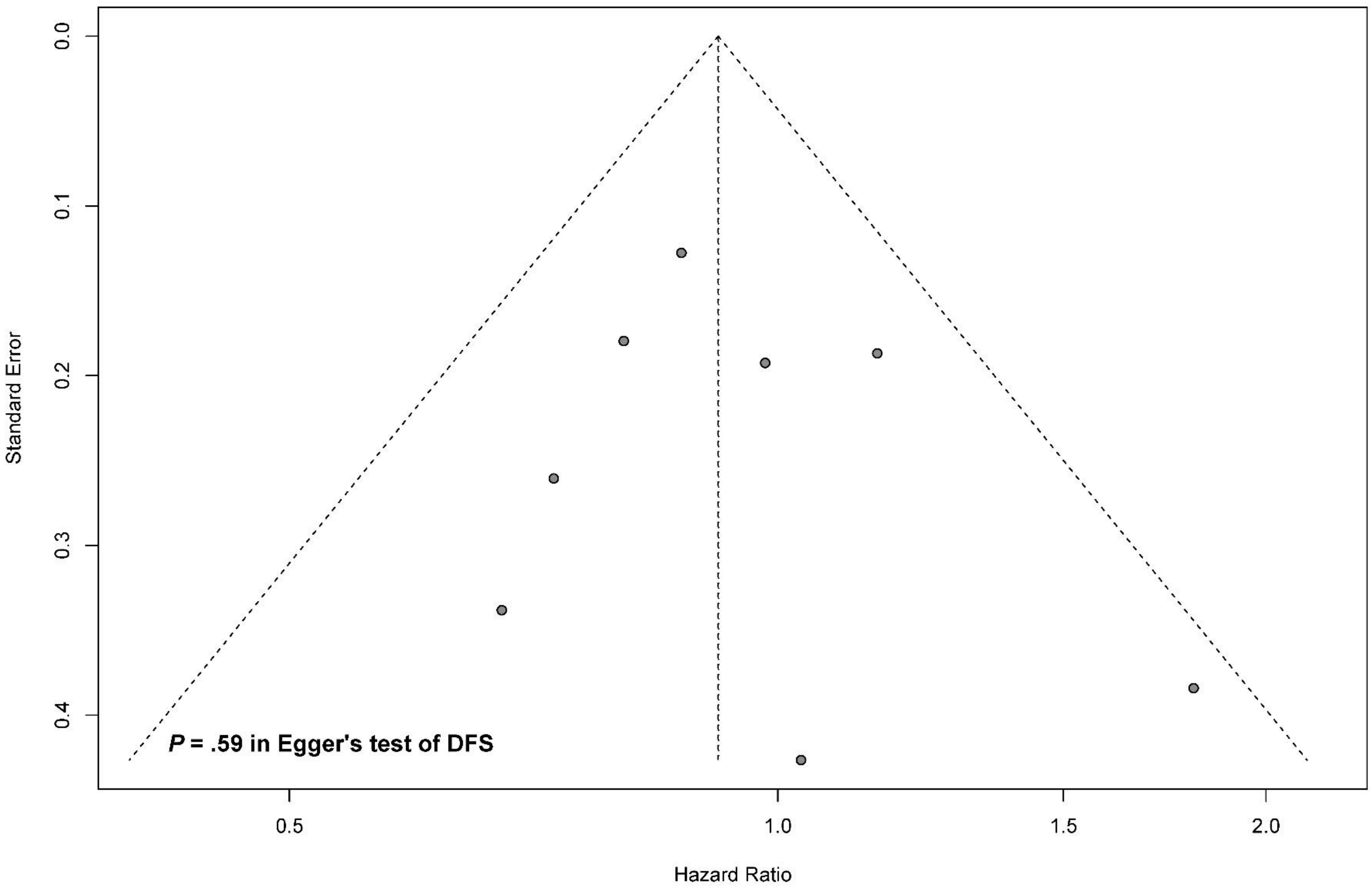

Abbreviations: DFS, disease-free survival.

© 2022 Kong M et al. *JAMA Network Open.*

eFigure 5. Funnel Plot and Egger's Test of OS

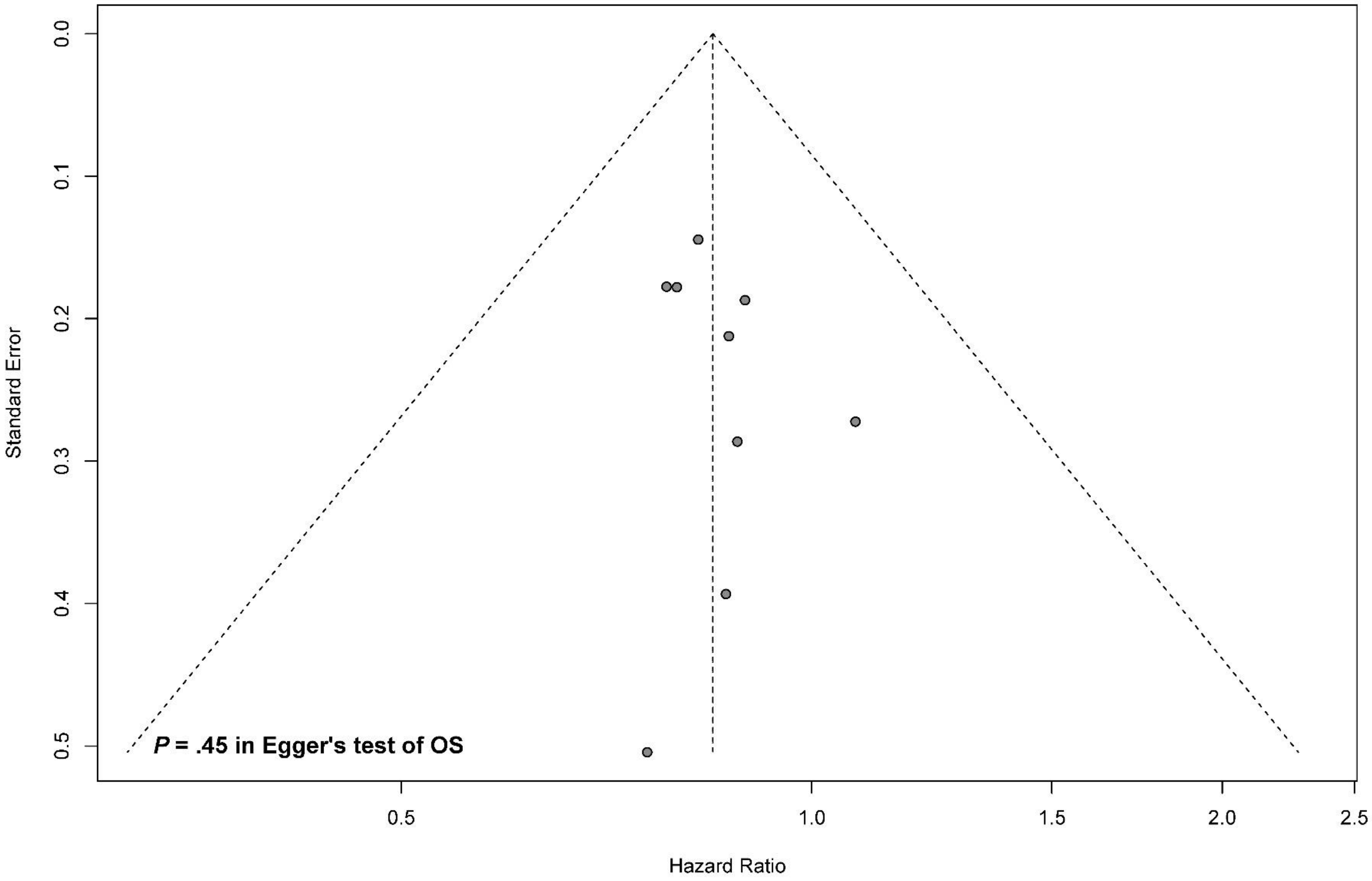

Abbreviations: OS, overall survival.

© 2022 Kong M et al. *JAMA Network Open.*

eAppendix 1. Supplementary Methods

A： Individual participant data extraction

The individual participant data (IPD) information of disease-free survival (DFS) and overall survival (OS) was extracted from the published Kaplan-Meier survival curves and then reconstructed. Briefly, graphical images of Kaplan-Meier curves from included studies were downloaded, and digitized to obtain the data coordinates (time and survival rate) using Engauge Digitizer (Version 12.1)[1]. And then, the extracted survival information was used to reconstruct individual-level time-to-event data by using the STATA *ipdfc* command, published by Wei et al[2]. The published risk tables were used to further calibrate the accuracy of the reconstructed IPD.

```
# reconstruct IPD using STATA ipdfc command
import excel "E:\Data of OS.xlsx", sheet("open for analysis") firstrow case(lower)
ipdfc, surv(s) tstart(ts) trisk(trisk) nrisk(nrisk) isotonic generate(t_ipd event_ipd) saving(temp0, replace)
clear
import excel "E:\ Data of OS.xlsx", sheet("Lapar for analysis") firstrow case(lower)
ipdfc, surv(s) tstart(ts) trisk(trisk) nrisk(nrisk) isotonic generate(t_ipd event_ipd) saving(temp1, replace)
use temp0, clear
gen byte arm = 0
append using temp1
replace arm = 1 if missing(arm)
label define ARM 0 "Open surgery" 1 "Laparoscopic surgery"
label values arm ARM
stset t_ipd, failure(event_ipd)
```

The reconstructed IPD was used to reconstruct the Kaplan-Meier survival curves by using the R *survival, survminer* and *ggplot2* packages. The risk tables were also generated. Hazard ratios (HRs) and 95% confidence intervals (CIs) between the two groups were calculated using the Cox proportional hazards model. We compared the reconstructed curves, risk tables, estimated HRs, and estimated 95% CIs with those in the original publications. The extraction was repeated if there were apparent discrepancies.

```
# generate Kaplan-Meier curves, risk tables, HRs and 95% CIs for each included study in R
library(survival)
library(survminer)
library(ggplot2)
x<-read.csv("E:/Data of OS for R.csv")
fit <- survfit(Surv(time, event) ~ arm, data=x)
ggsurvplot(fit,
           risk.table=TRUE
           ...)
res_cox <- coxph(Surv(time, event) ~ arm, data=x)
summary(res_cox)
```

B: Reconstruction of survival curves and estimation of survival rates for the whole populations

The reconstructed IPD from all the included RCTs was combined, and Kaplan-Meier curves and risk tables of DFS and OS were separately generated for the whole included population using the following codes in R:

```
# generate Kaplan-Meier curves and risk tables for the whole population in R
library(survival)
```

```
library(survminer)
library(ggplot2)
x<-read.csv("E:/Data of OS all for R.csv")
fit <- survfit(Surv(time, event) ~ arm, data=x)
ggsurvplot(fit,
           risk.table=TRUE,
           ...)
```

The 5-year survival rates and 95% CIs were estimated using Kaplan–Meier method by the STATA *sts list* command.

```
# list the estimated survivor and related functions using STATA sts list command
clear
use "E:\ Data of OS all.dta"
sts list if arm==1
sts list if arm==0
```

C: One-stage IPD meta-analysis

For the primary analysis, we did a one-stage IPD meta-analysis using Cox-based shared-frailty model[3]. The shared-frailty approach is the survival-data analog to regression models with random effects, and assumes that the hazards within each study are proportional to the same common baseline hazard function. The model is designed to account for differences in unmeasured covariates between trials. The analysis was performed using STATA *stcox* command, or R *survival* package. We assessed between-study heterogeneity using the estimated between-study variance in random effects (τ^2), which was calculated by the following formula[4]: $\tau^2 = [\theta/(\theta+2)]^2$, ,where θ is variance of the frailty distribution.

```
# One-stage IPD meta-analysis (Cox-based shared-frailty model) using STATA stcox command
clear
use "E:\ Data of OS all.dta"
stcox arm, shared(studyid)
```

```
# One-stage IPD meta-analysis (Cox-based shared-frailty model) in R
library(survival)
x<-read.csv("E:/Data of OS all for R.csv")
res_cox_one <- coxph(Surv(time, event) ~ arm+frailty(studyid), data=x)
summary(res_cox_one)
```

D: **Two-stage IPD meta-analysis**

The estimated HRs and CIs of each of the included studies were pooled using a fixed-effects model or a random-effects model based on the degree of heterogeneity. The inverse variance method was used. *P* values < 0.05 were required for the overall HRs to be statistically significant. Heterogeneity was assessed using the I^2 and chi-squared measures. Potential publication biases were analyzed using funnel plot and Egger's test. A *P* value of Egger's test <0.10 suggested the existence of potential publication bias The R *meta* package was used as the following codes:

```
# Two-stage IPD meta-analysis in R
library(meta)
x <- read.csv("E:/OS all for two step.CSV")
mg1 <- metagen(logHR, selogHR,
```

© 2022 Kong M et al. *JAMA Network Open.*

```
                    studlab=paste(study, year), data=x,
                    sm="HR")
# forest plot
forest(mg1,
        ...)
# funnel plot
funnel(mg1)
# Egger's test
metabias(mg1,method.bias="linreg",plotit=T,k.min=5)
```

eReferences.

1. Mark Mitchell BMaTWea. Engauge Digitizer Software." Webpage: http://markummitchell.github.io/engauge-digitizer, Last Accessed: December 24, 2020.
2. Wei Y, Royston P. Reconstructing time-to-event data from published Kaplan-Meier curves. *The Stata journal.* 2017;17(4):786-802.
3. de Jong VMT, Moons KGM, Riley RD, et al. Individual participant data meta-analysis of intervention studies with time-to-event outcomes: A review of the methodology and an applied example. *Research synthesis methods.* 2020;11(2):148-168.
4. Munda M, Legrand C. Adjusting for centre heterogeneity in multicentre clinical trials with a time-to-event outcome. *Pharmaceutical statistics.* 2014;13(2):145-152.

© 2022 Kong M et al. *JAMA Network Open.*

eAppendix 2. Reconstructed Survival Curve and Side-by-Side Comparison With the Original Curve for Each Included Study*

*The top curve in each page was the reconstructed one, and the bottom curve was the original published.

Abbreviations: DFS, disease-free survival; OS, overall survival.

© 2022 Kong M et al. *JAMA Network Open.*

Braga, 2007-DFS

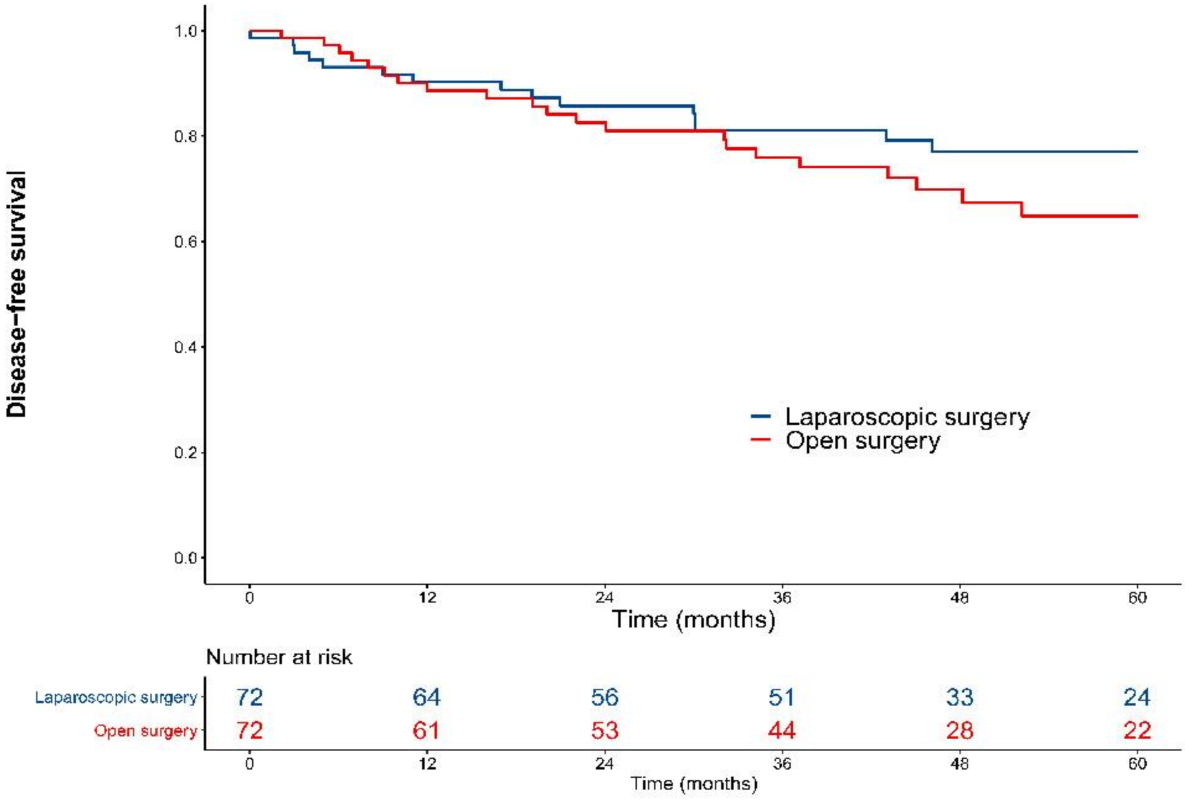

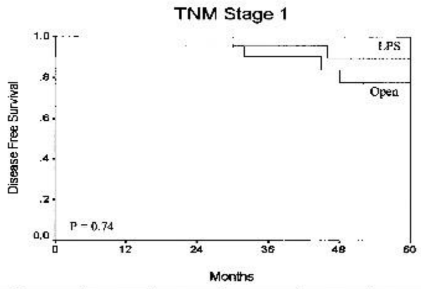

LPS	25	25	23	23	19	14
Open	24	24	21	16	12	10

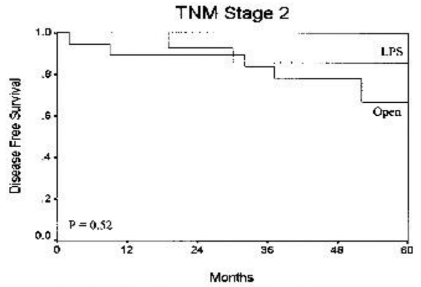

LPS	16	16	13	11	2	2
Open	19	17	17	15	7	5

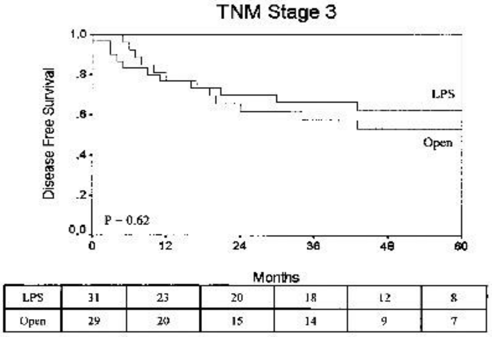

LPS	31	23	20	18	12	8
Open	29	20	15	14	9	7

© 2022 Kong M et al. *JAMA Network Open.*

Braga, 2007-OS

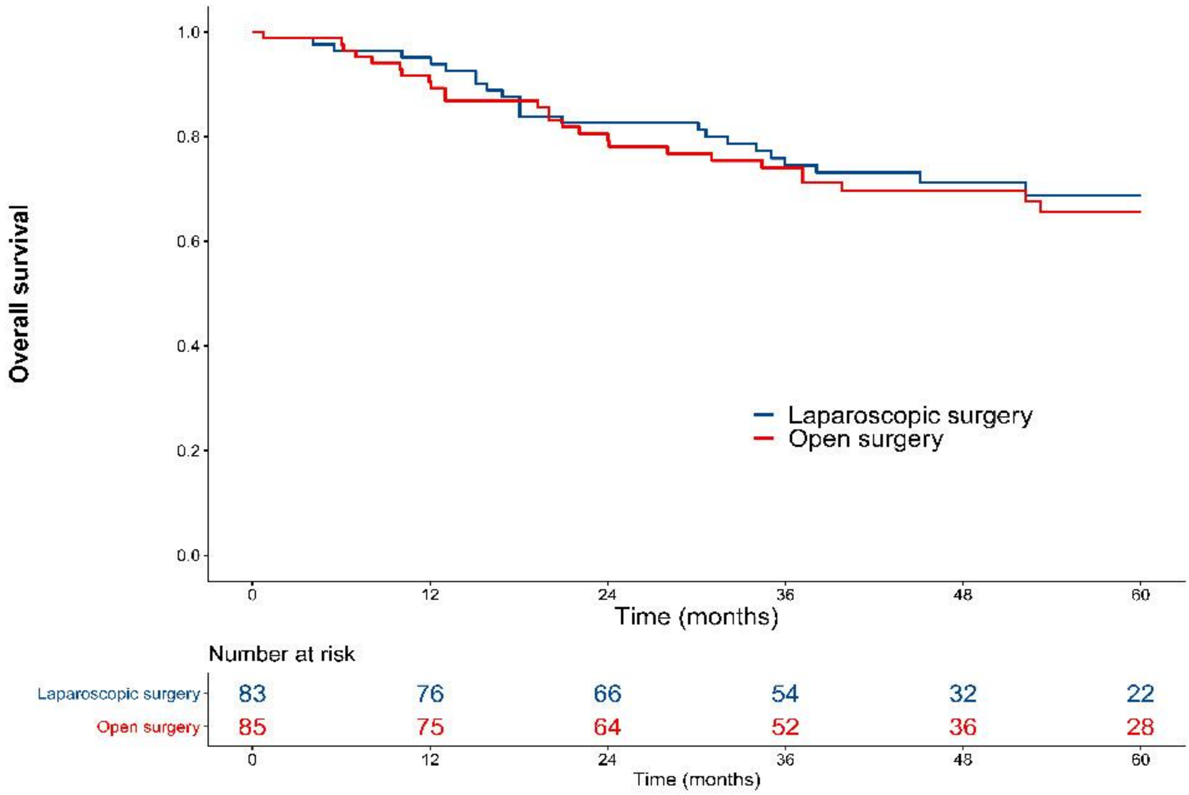

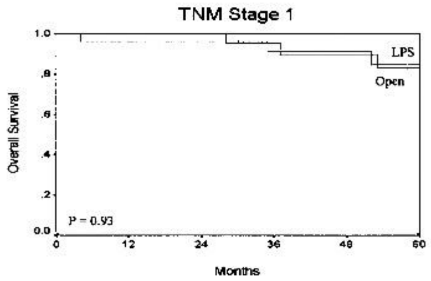

LPS	25	24	24	19	15	10
Open	24	24	24	18	15	11

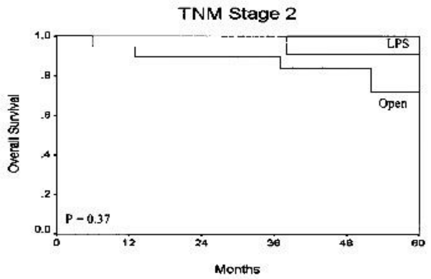

LPS	16	16	16	15	4	3
Open	19	18	17	16	7	5

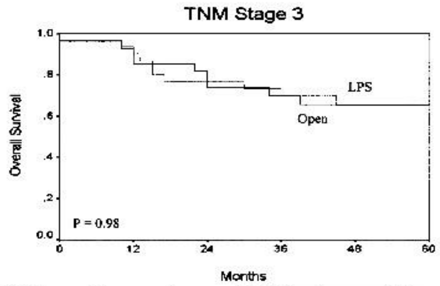

LPS	31	27	22	19	12	8
Open	29	24	19	16	12	10

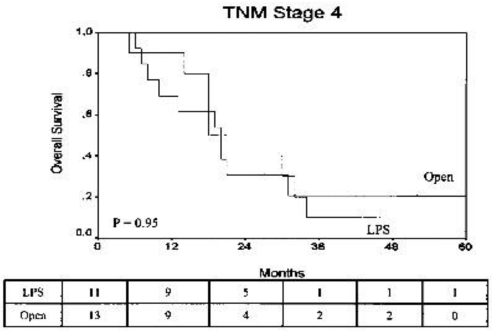

LPS	11	9	5	1	1	1
Open	13	9	4	2	2	0

© 2022 Kong M et al. *JAMA Network Open.*

Lujan, 2009-DFS

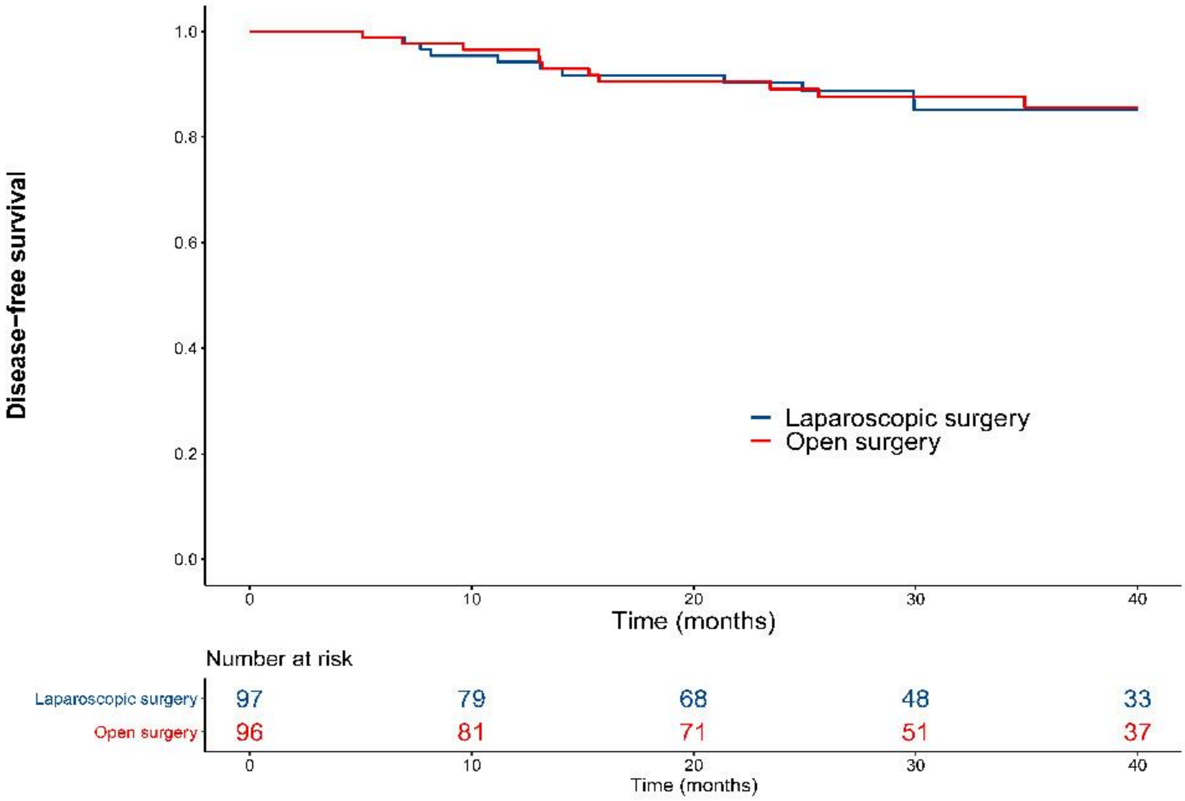

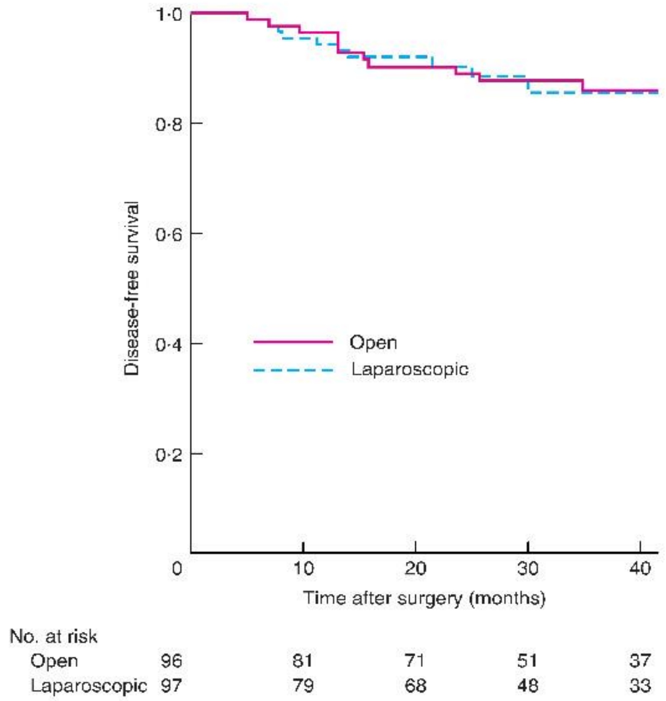

Fig. 3 Kaplan–Meier survival curves comparing disease-free survival in open and laparoscopic groups

© 2022 Kong M et al. *JAMA Network Open.*

Lujan, 2009-OS

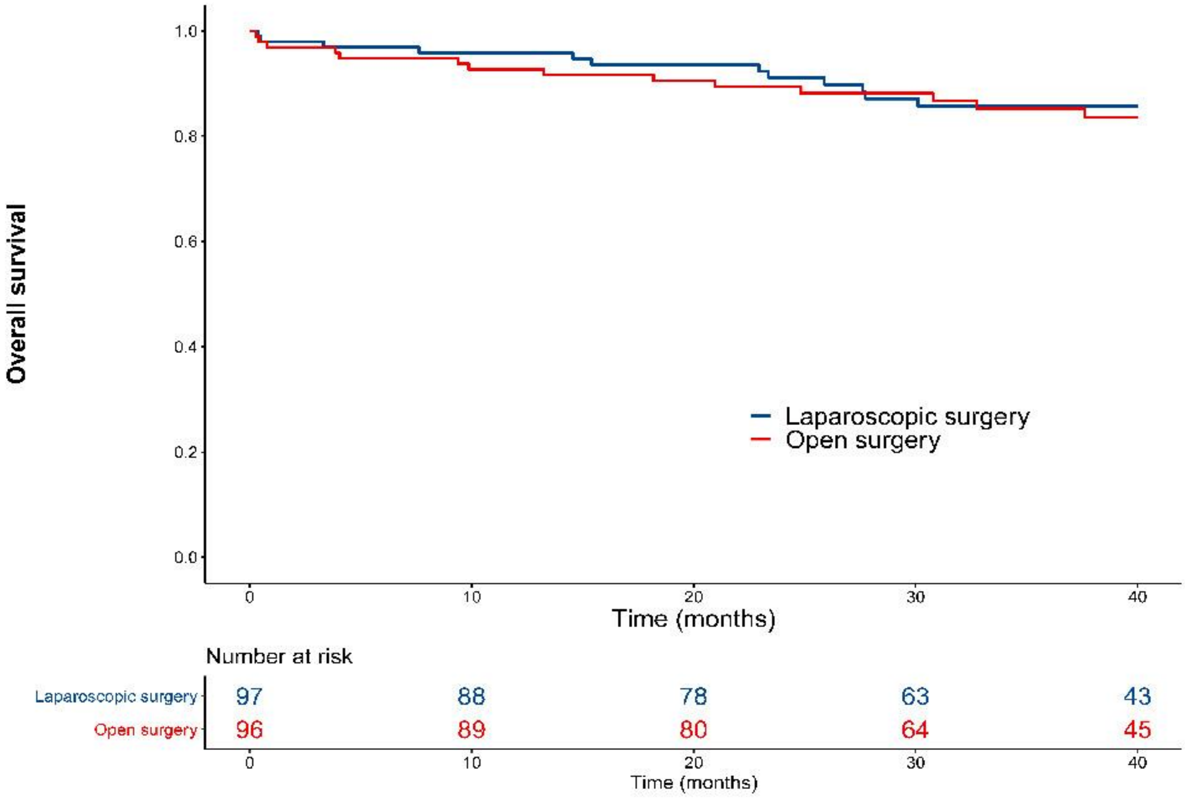

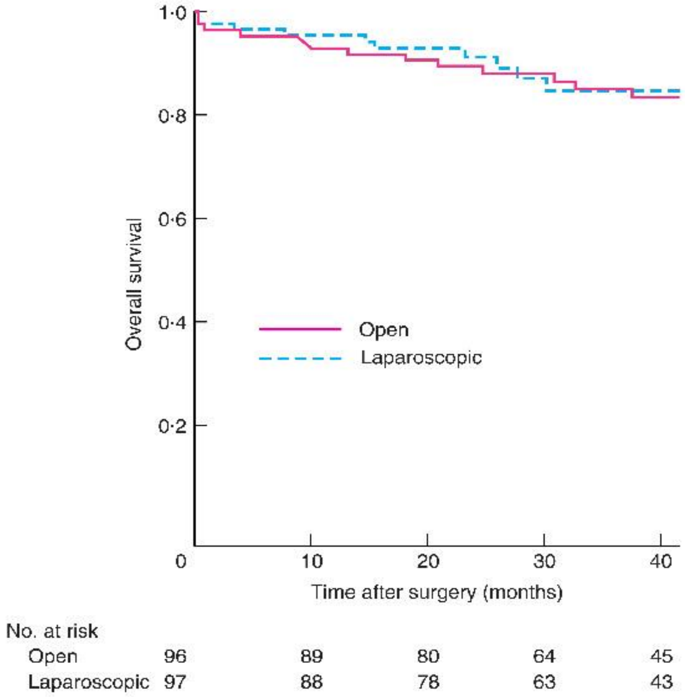

Fig. 4 Kaplan–Meier survival curves comparing overall survival in open and laparoscopic groups

© 2022 Kong M et al. *JAMA Network Open.*

Liang, 2011-OS

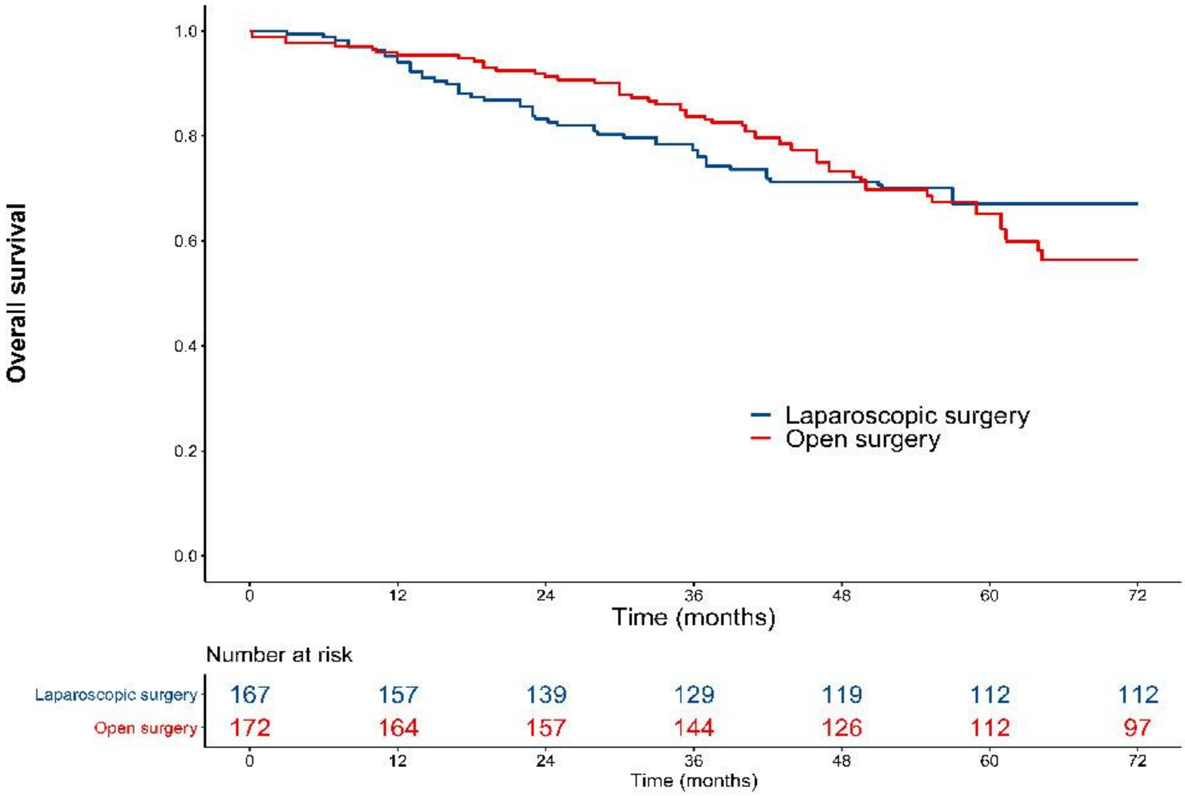

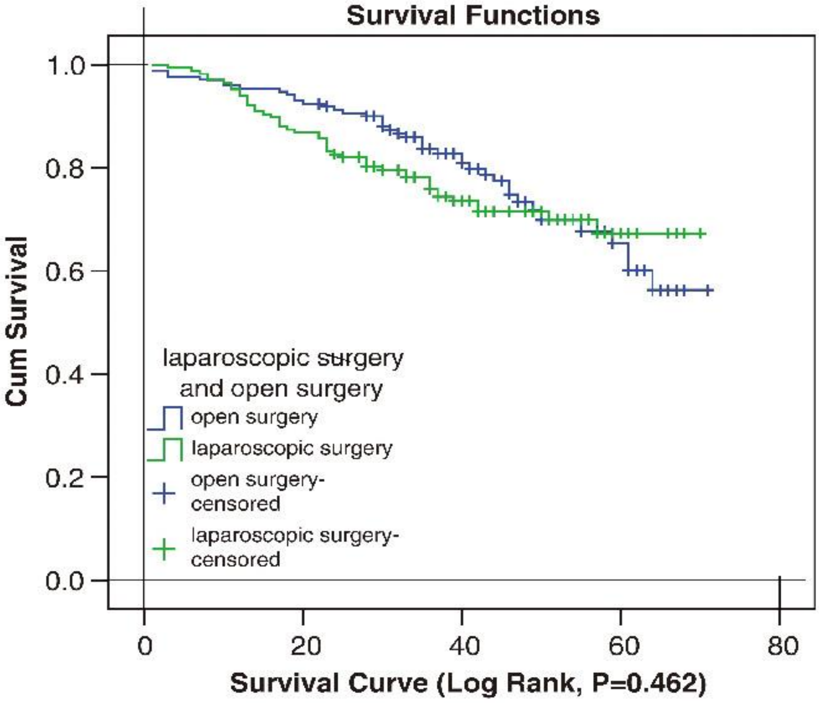

FIG. 2. Survival curve.

© 2022 Kong M et al. *JAMA Network Open*.

CLASICC, 2013-OS

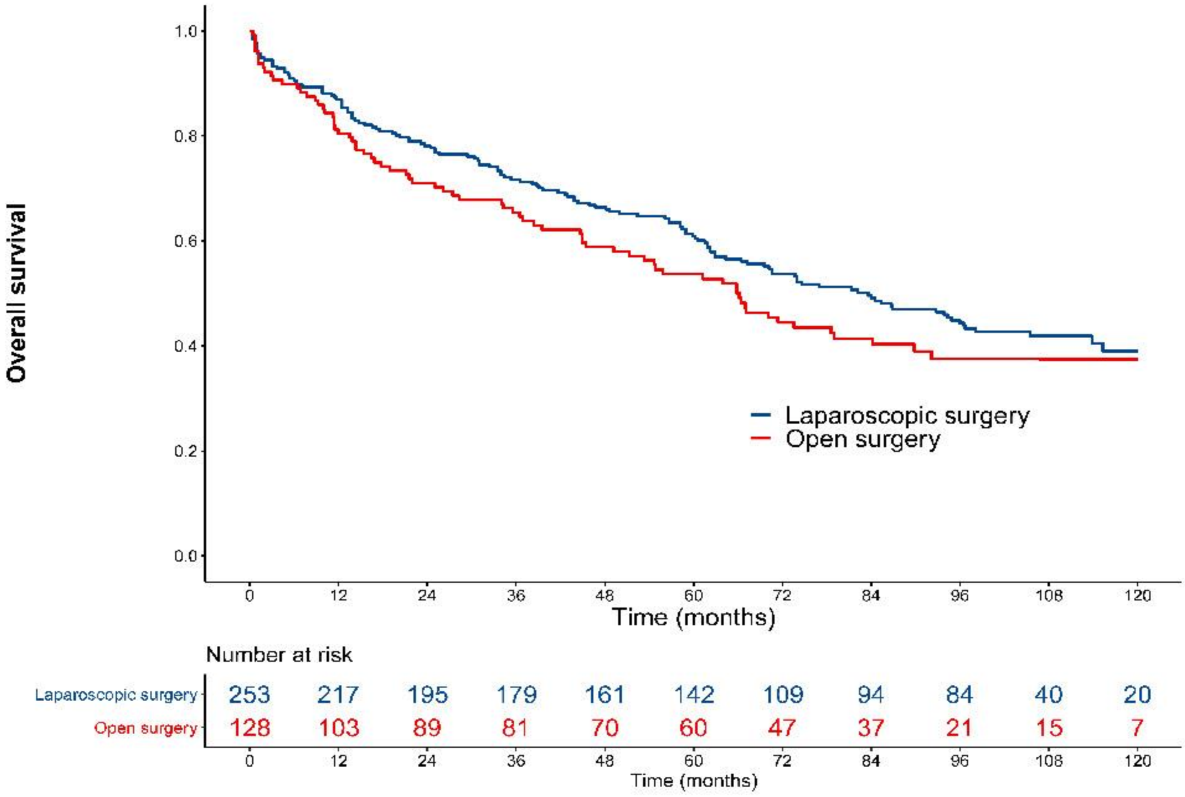

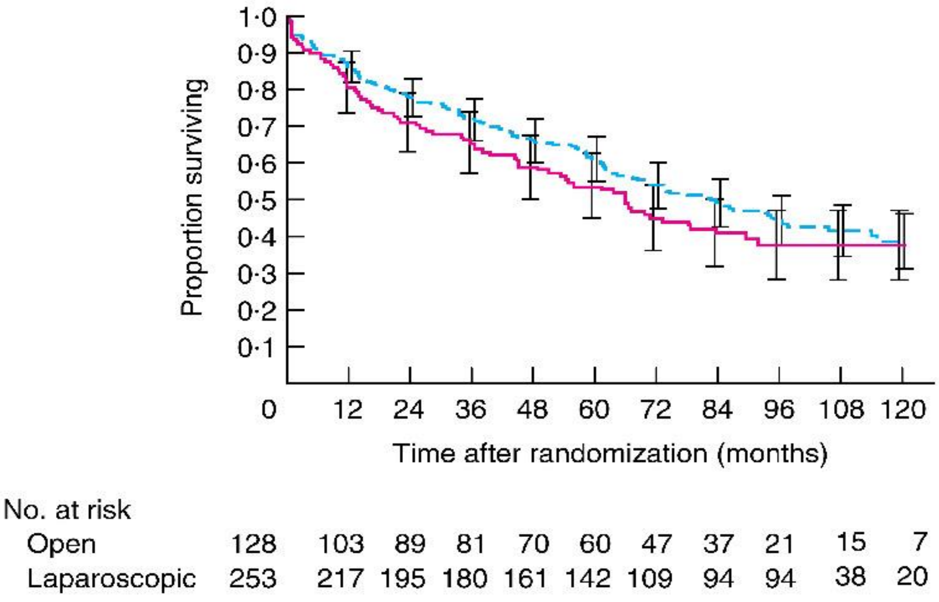

b Rectal cancer

Fig. 2 Overall survival by randomized procedure for **a** colonic and **b** rectal cancer. Error bars represent 95 per cent confidence

© 2022 Kong M et al. *JAMA Network Open.*

Ng, 2014-DFS

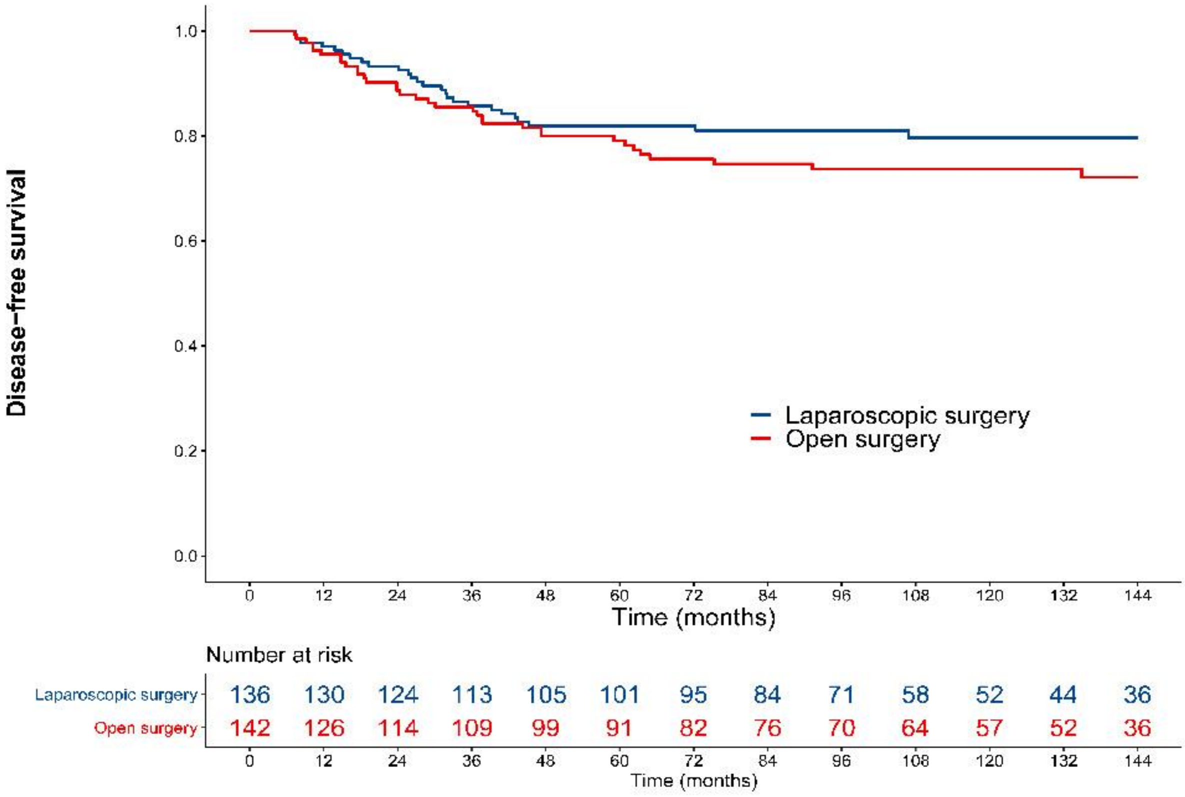

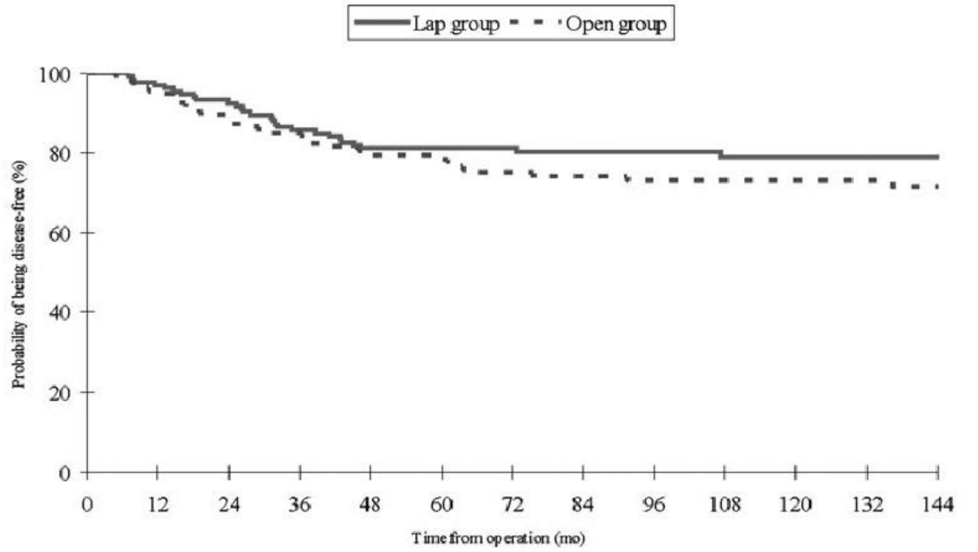

Time from operation (mo)

No. at risk	0	12	24	36	48	60	72	84	96	108	120	132	144
Lap group	136	130	124	113	105	101	95	84	71	58	52	44	36
Open group	142	126	114	109	99	91	82	76	70	64	57	52	36

FIGURE 3. Probability of being disease-free for all patients ($P = 0.218$, log-rank test).

© 2022 Kong M et al. *JAMA Network Open.*

Ng, 2014-OS

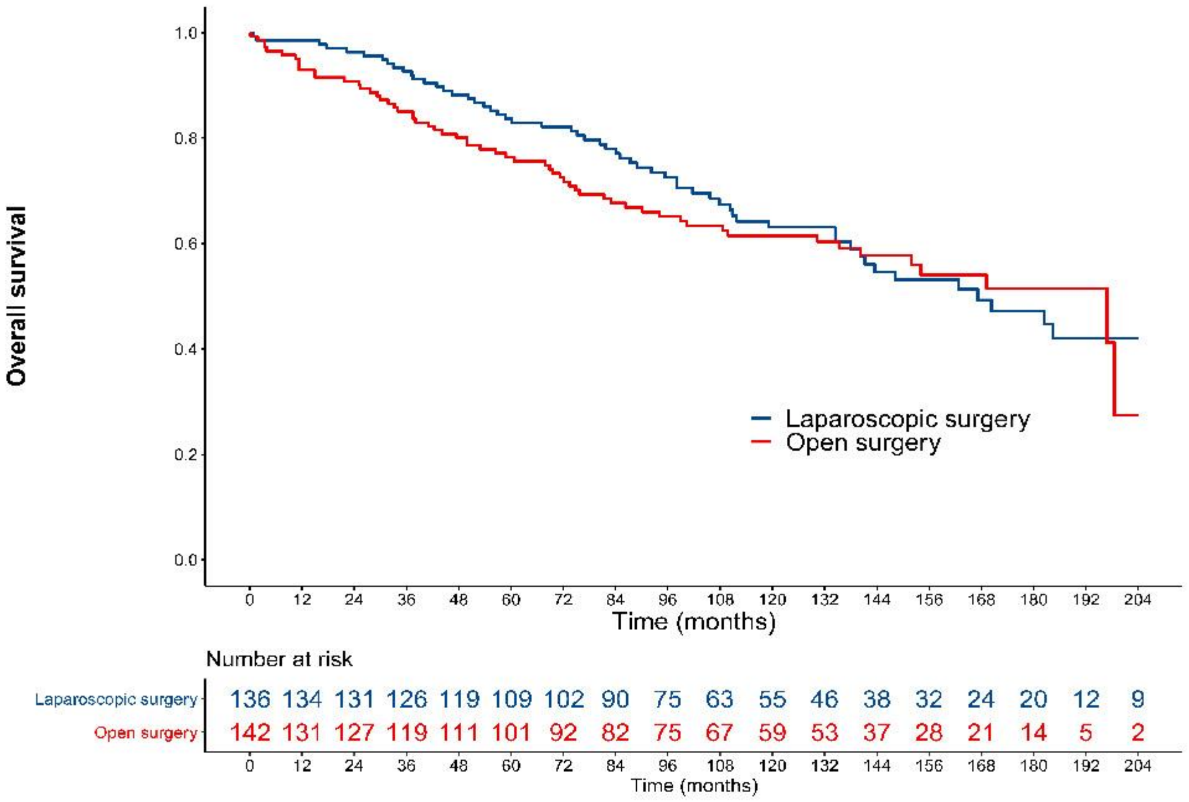

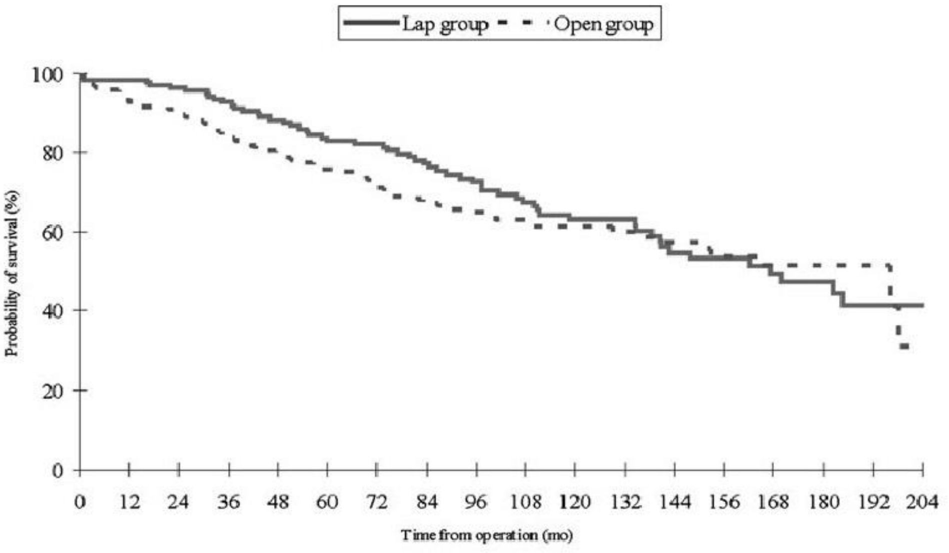

Time from operation (mo)

No. at risk	0	12	24	36	48	60	72	84	96	108	120	132	144	156	168	180	192	204
Lap group	136	134	131	126	119	109	102	90	75	63	55	46	38	32	24	20	12	9
Open group	142	131	127	119	111	101	92	82	75	67	59	53	37	28	21	14	5	2

FIGURE 1. Overall survival for all patients ($P = 0.505$, log-rank test).

© 2022 Kong M et al. *JAMA Network Open.*

COLOR II, 2015-DFS

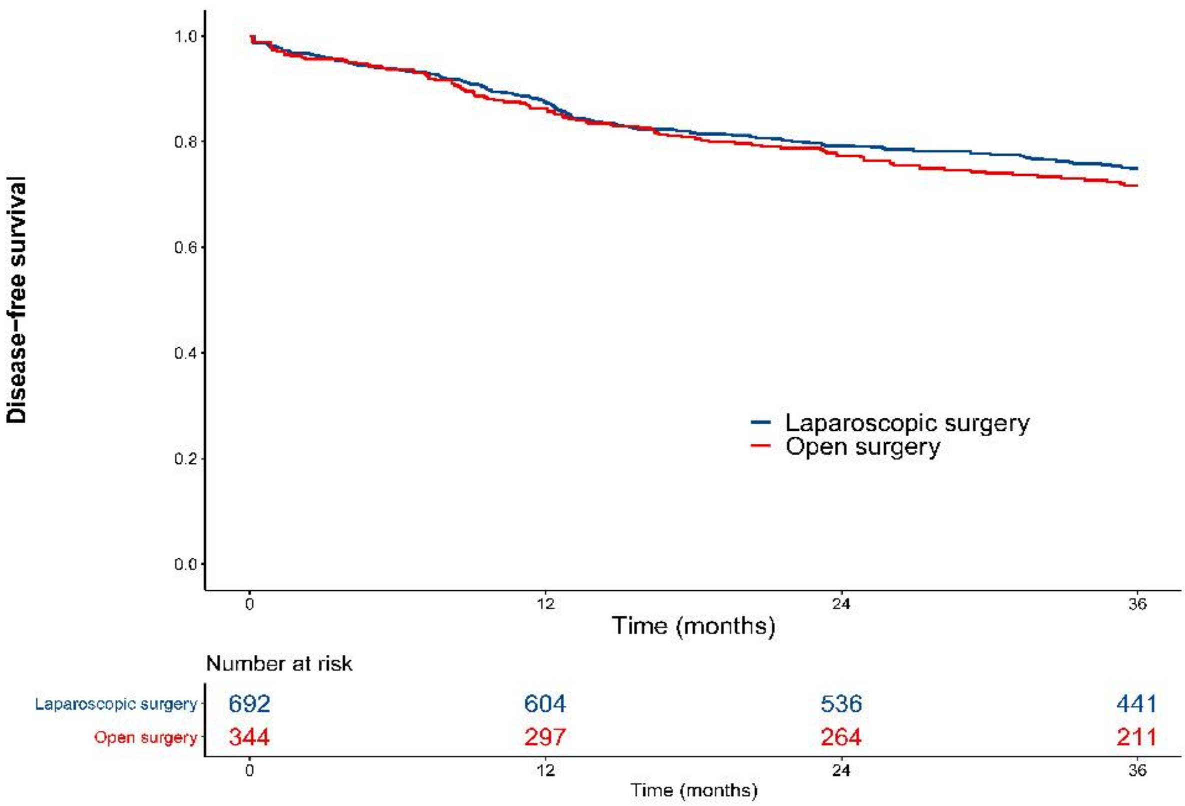

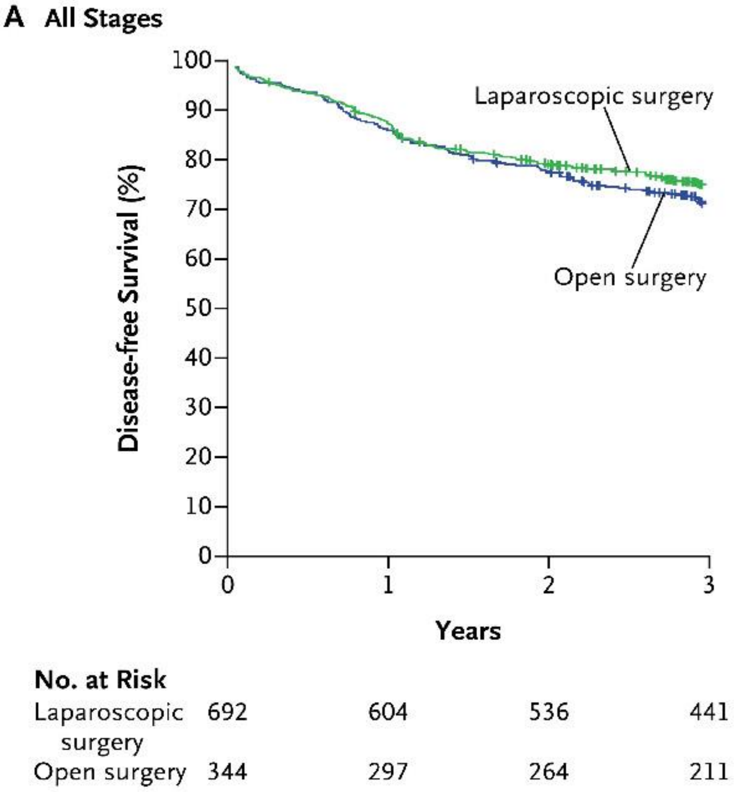

© 2022 Kong M et al. *JAMA Network Open.*

COLOR II, 2015-OS

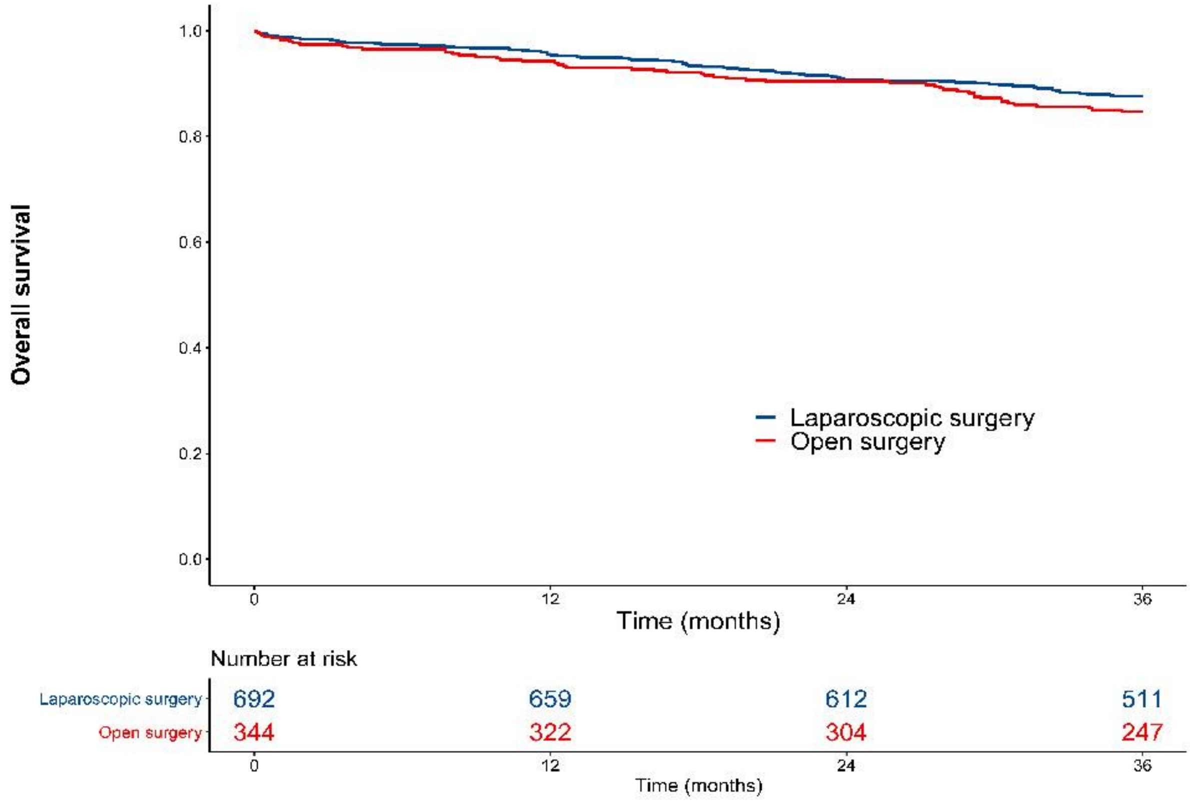

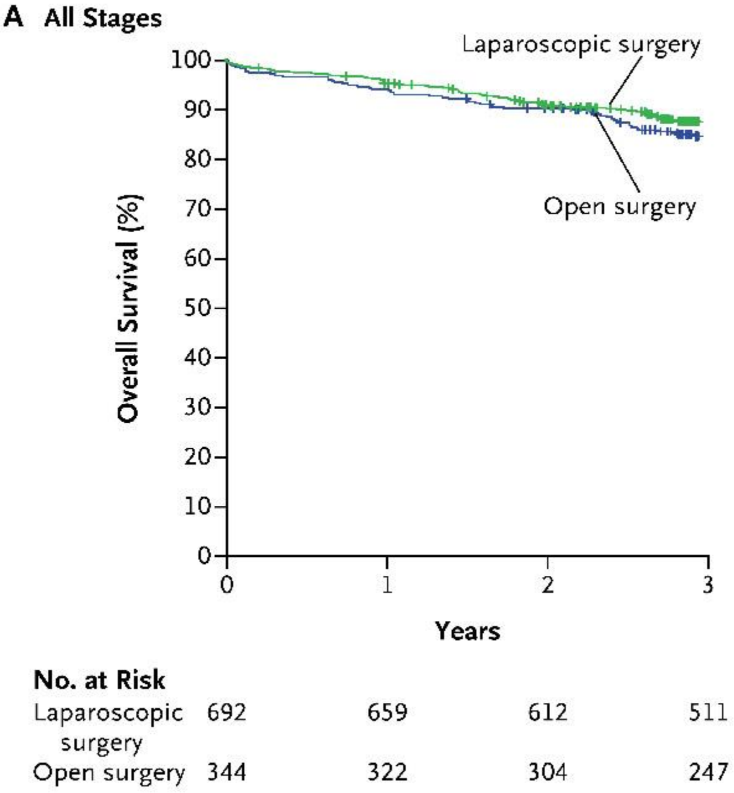

© 2022 Kong M et al. *JAMA Network Open*.

ACOSOG Z6051, 2019-DFS

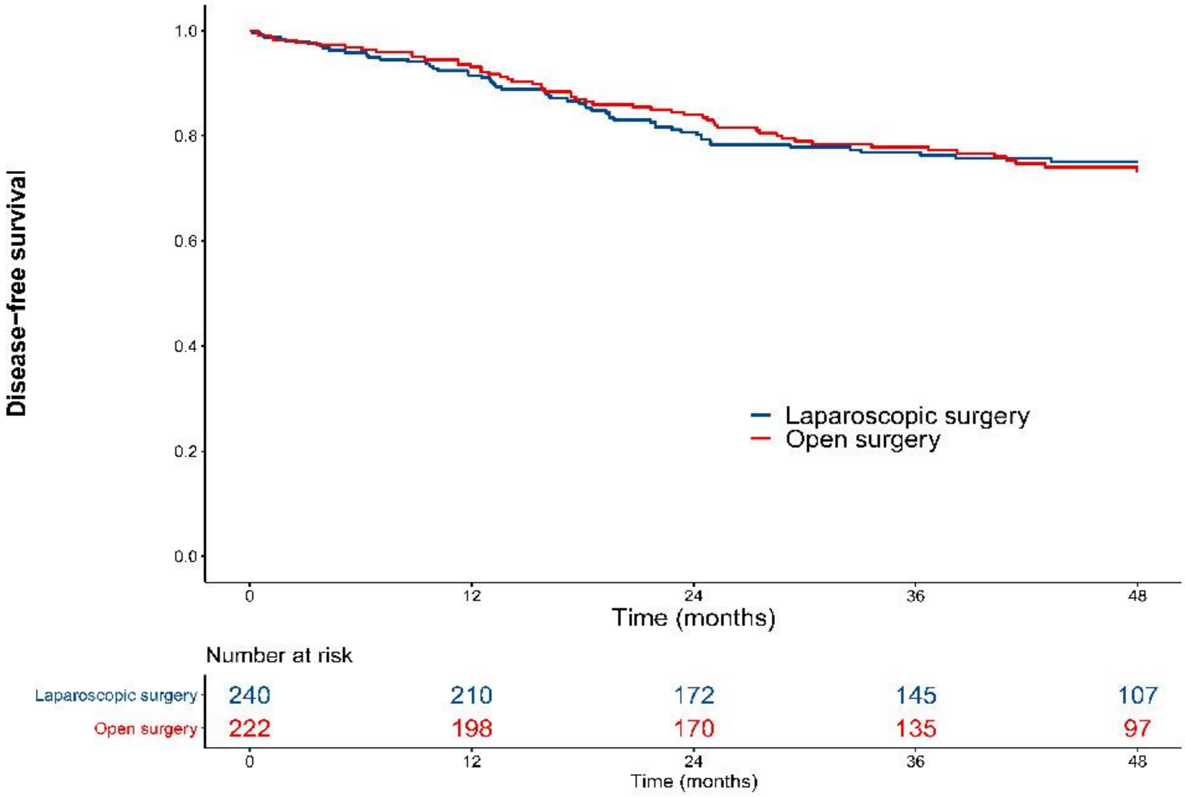

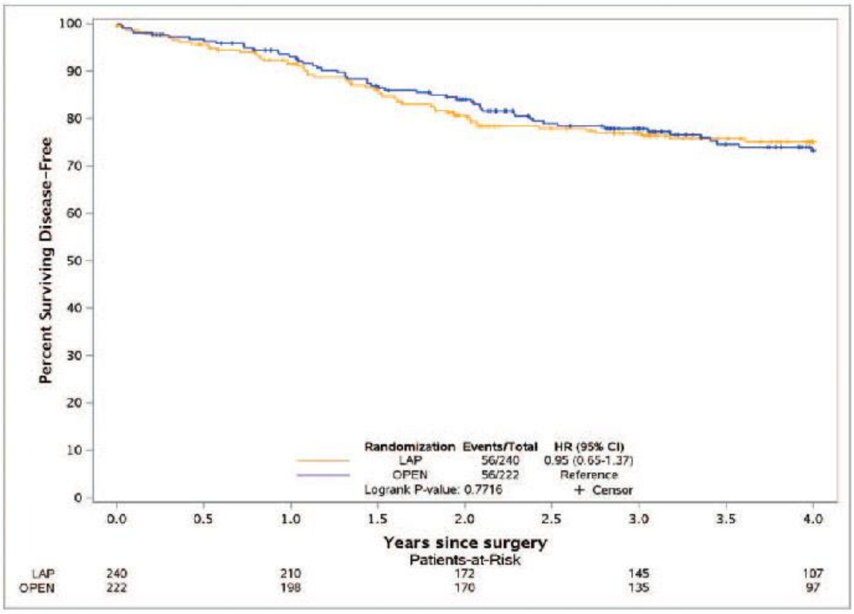

FIGURE 1. Disease-free survival of laparoscopic resection compared to open resection of stage II to III rectal cancer.

© 2022 Kong M et al. *JAMA Network Open*.

ALa CaRT, 2019-DFS

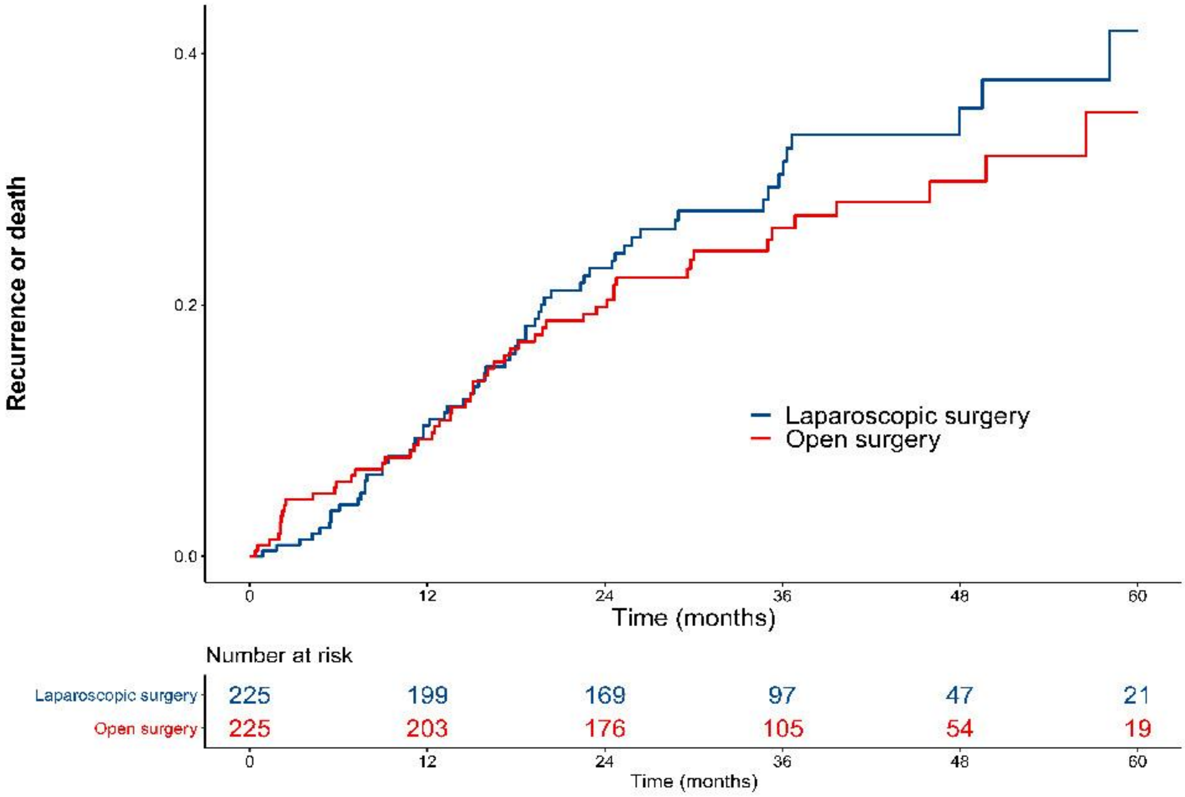

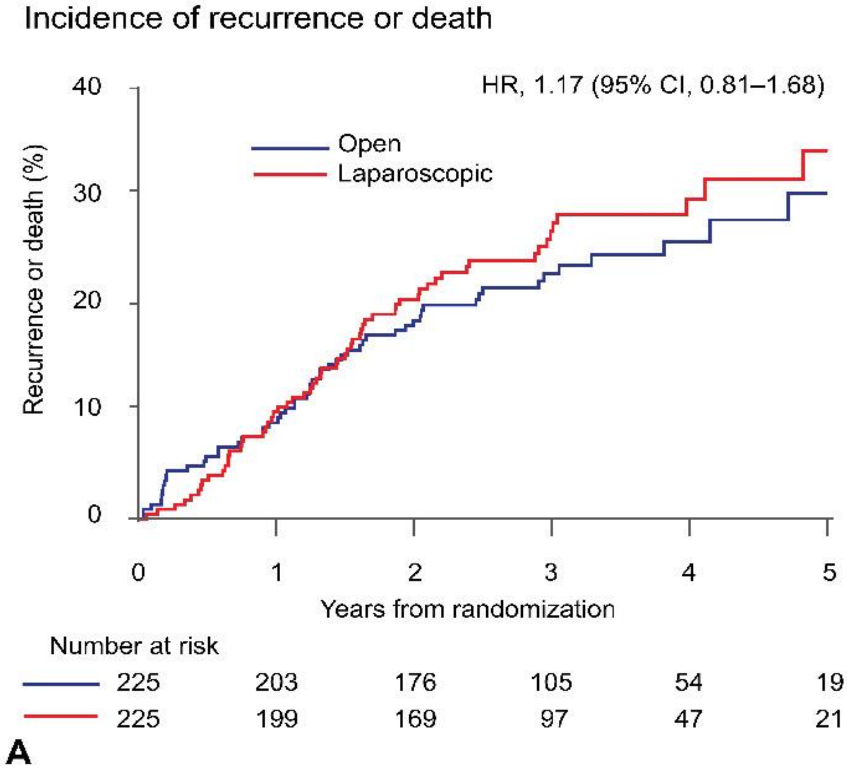

© 2022 Kong M et al. *JAMA Network Open*.

ALa CaRT, 2019-OS

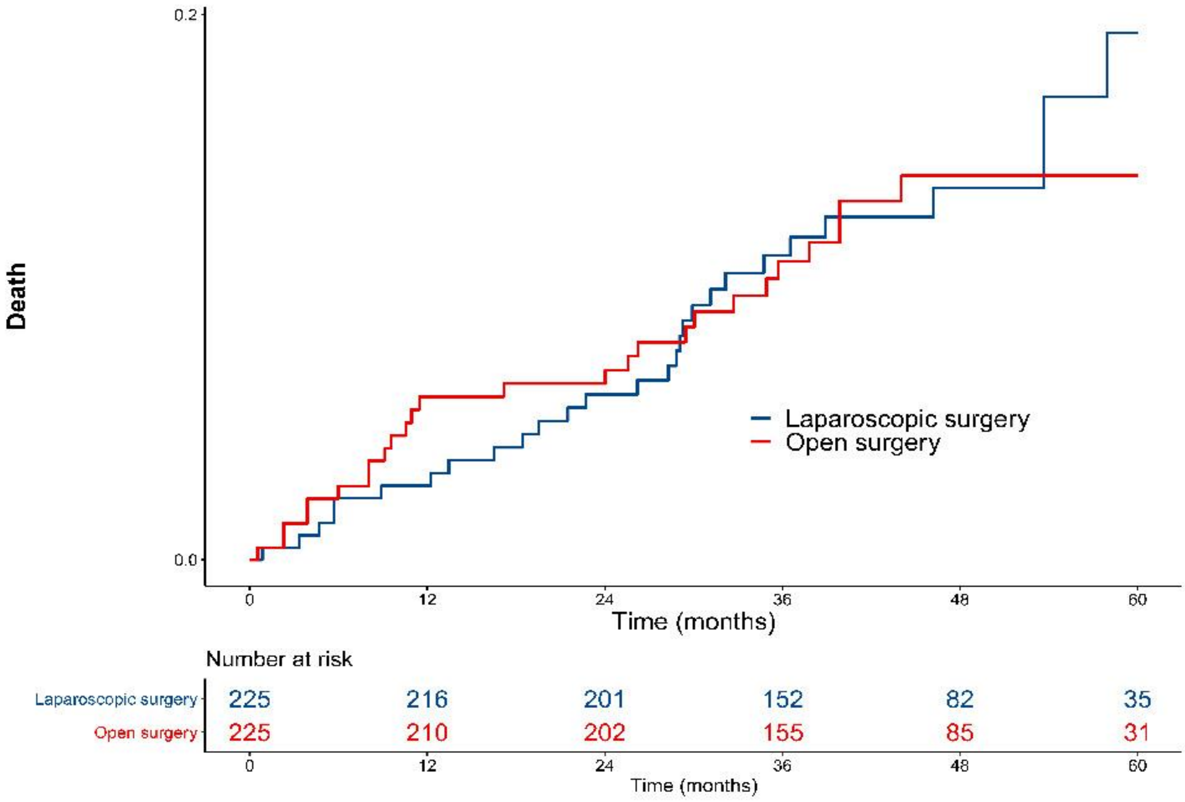

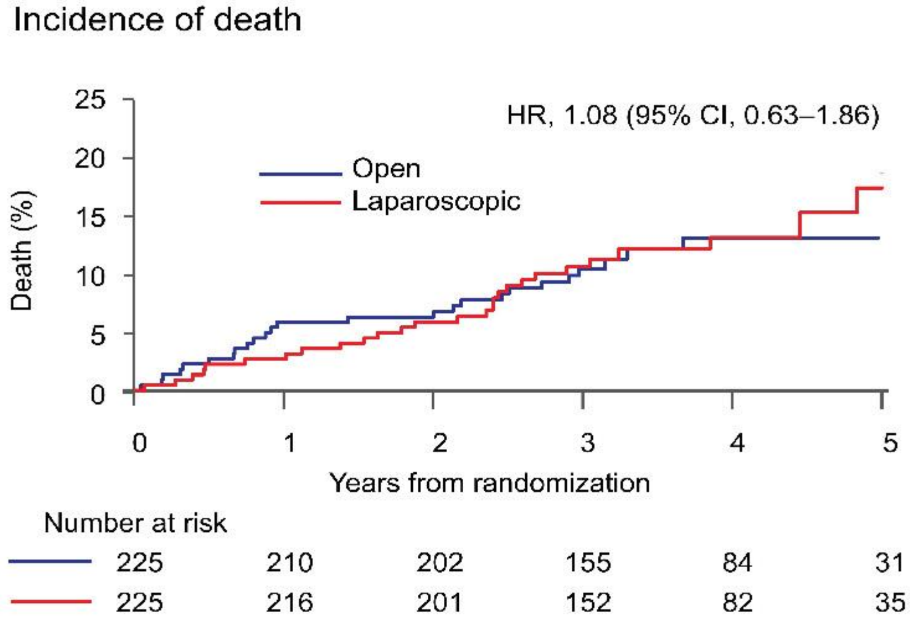

B

FIGURE 3. (A) Cumulative incidence of recurrence or death; (B) cumulative incidence of death by surgical group.

© 2022 Kong M et al. *JAMA Network Open.*

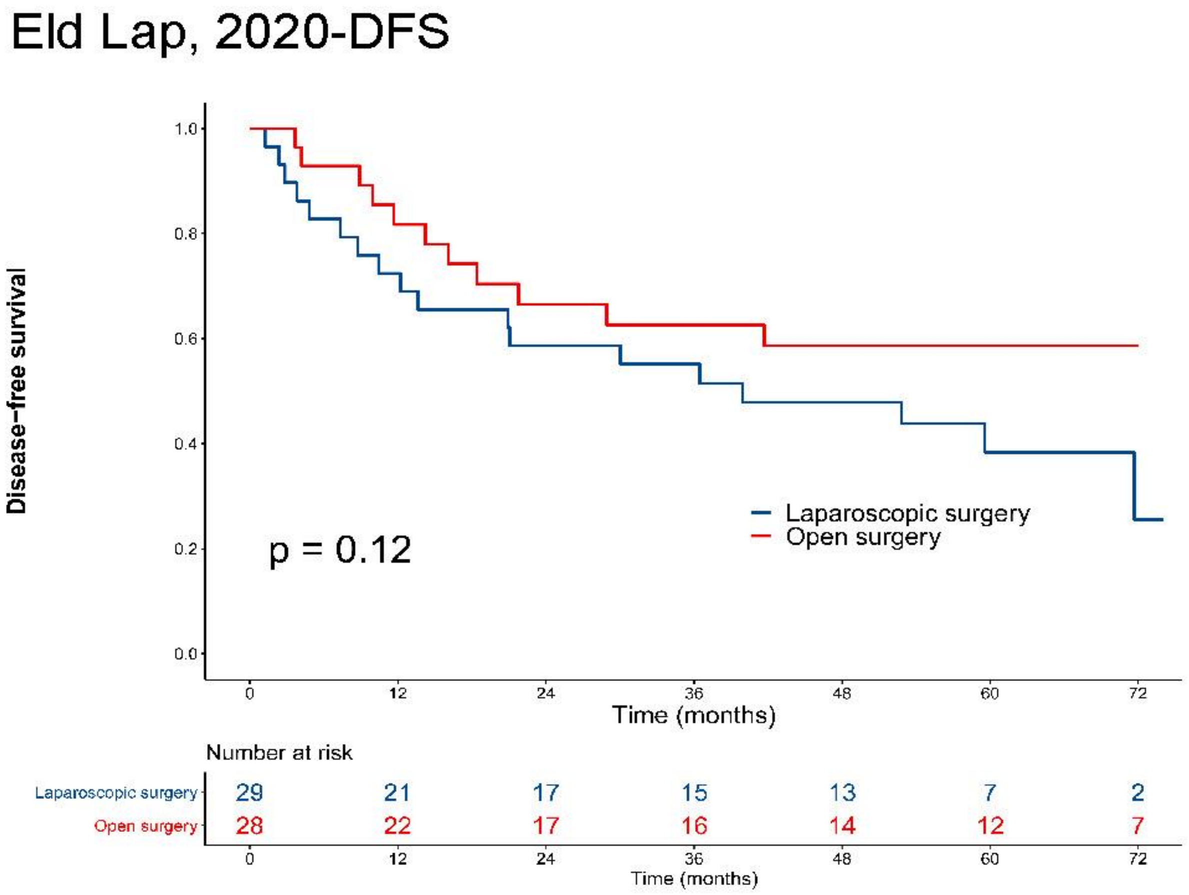

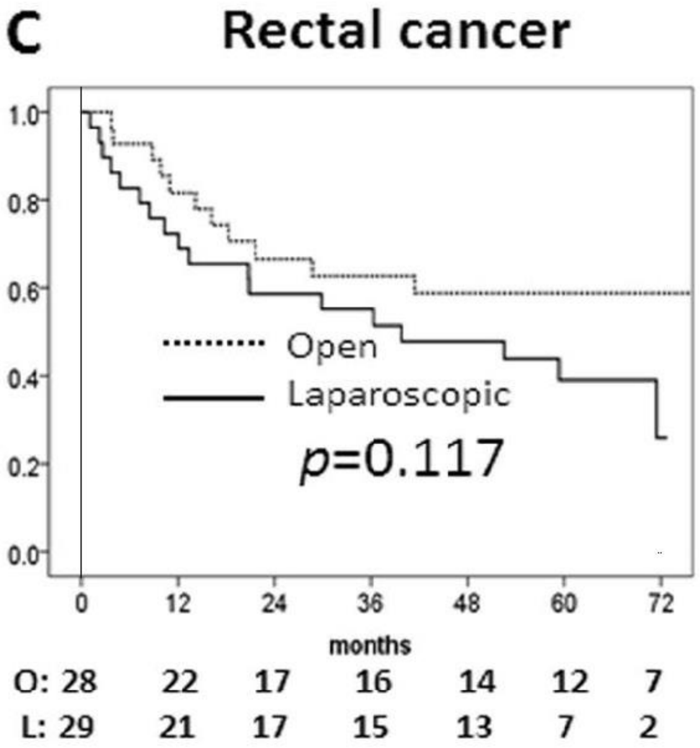

© 2022 Kong M et al. *JAMA Network Open*.

Eld Lap, 2020-OS

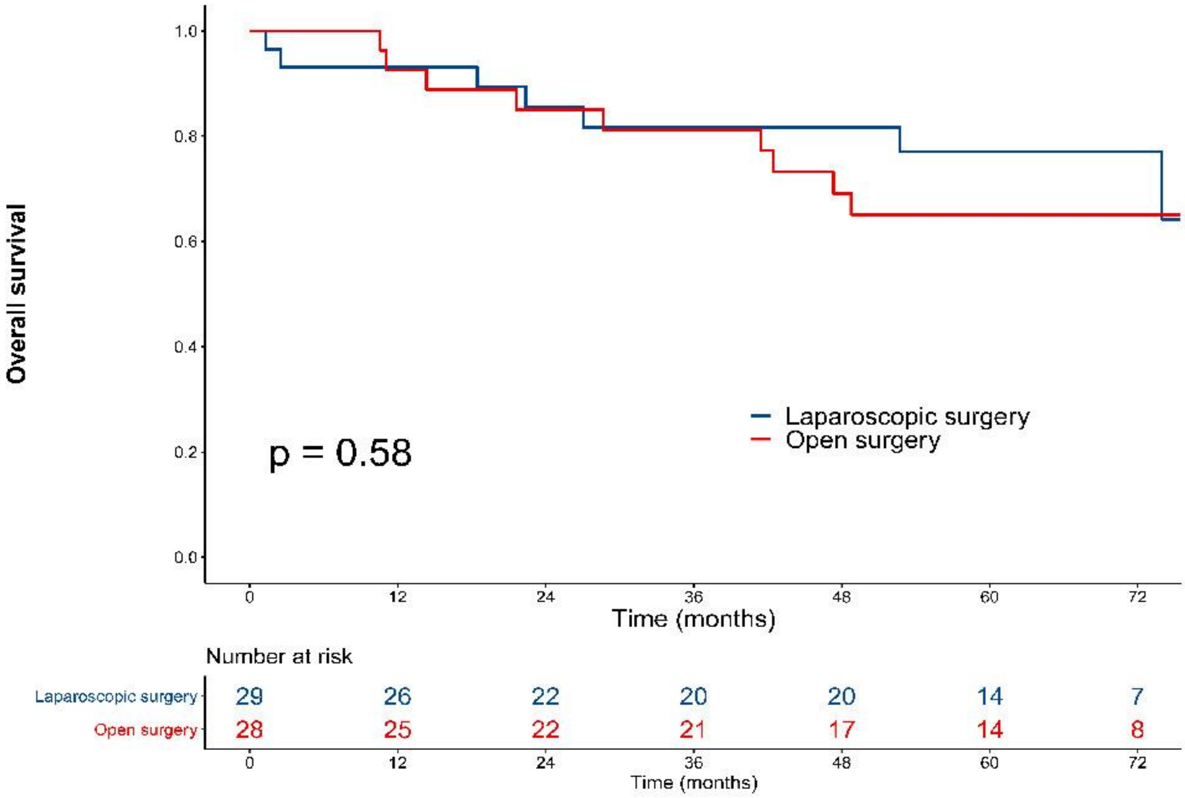

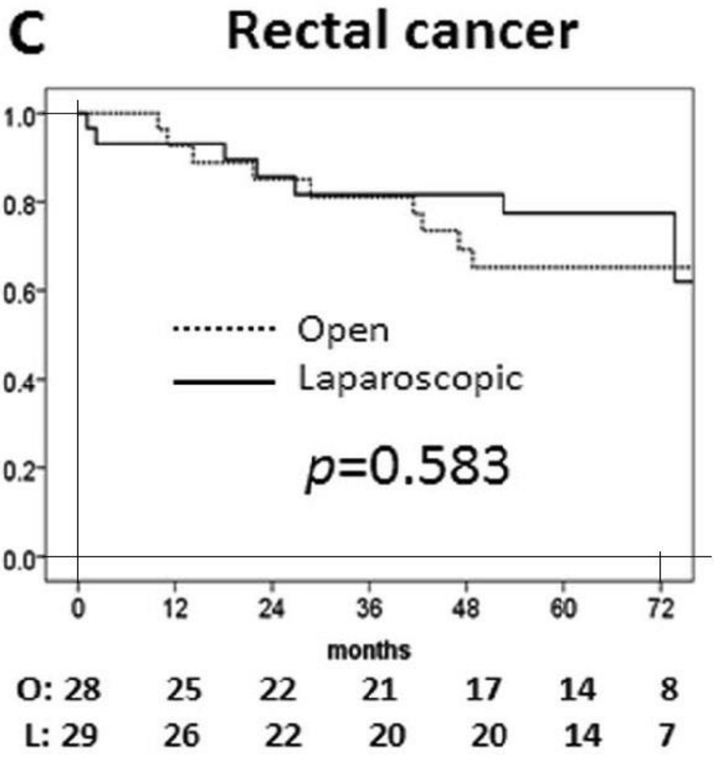

© 2022 Kong M et al. *JAMA Network Open*.

COREAN, 2021-DFS

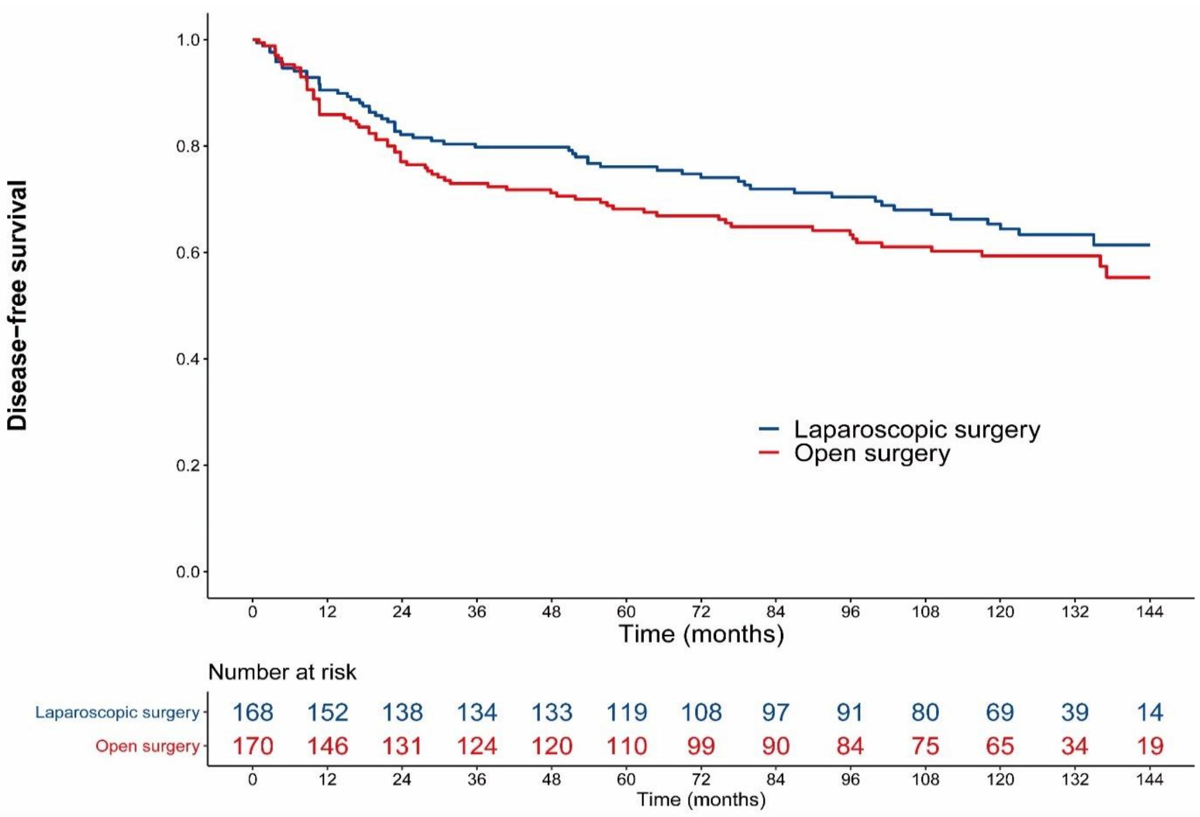

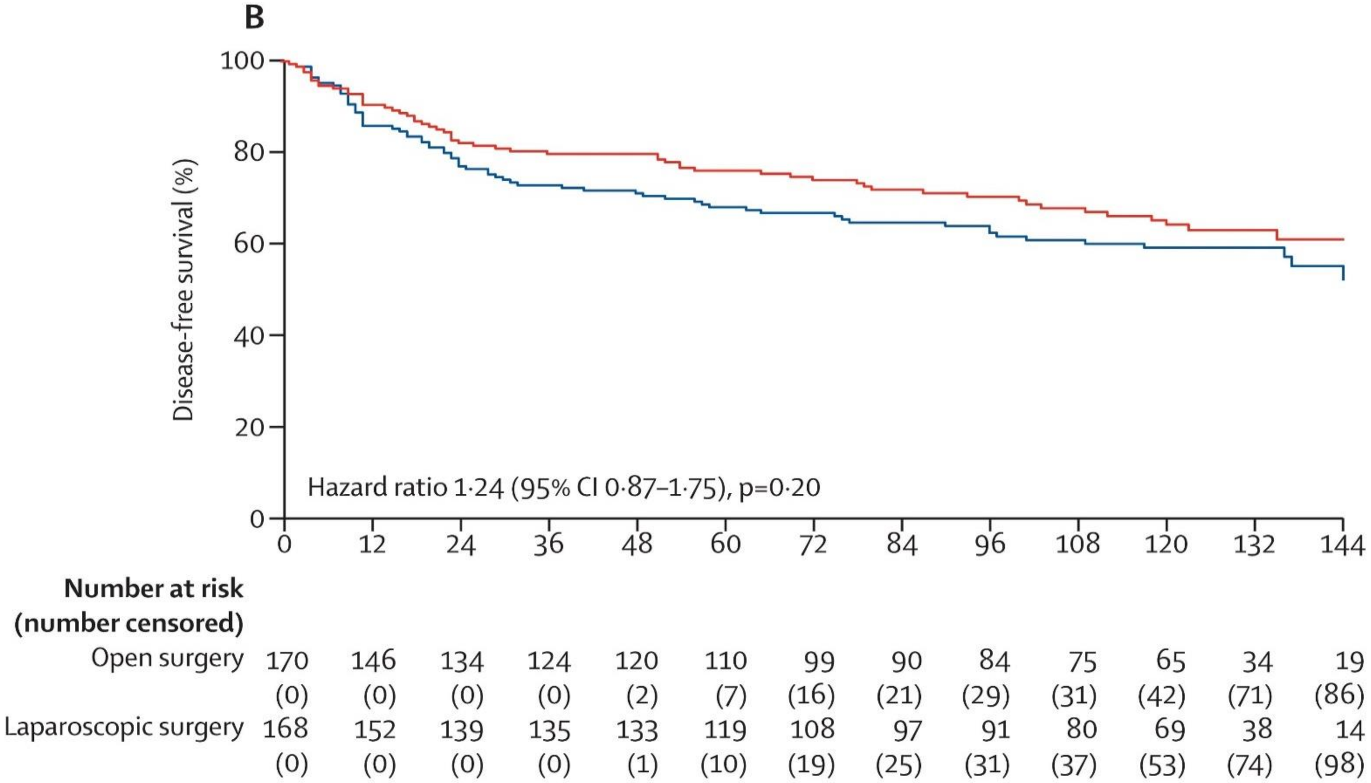

© 2022 Kong M et al. *JAMA Network Open.*

COREAN, 2021-OS

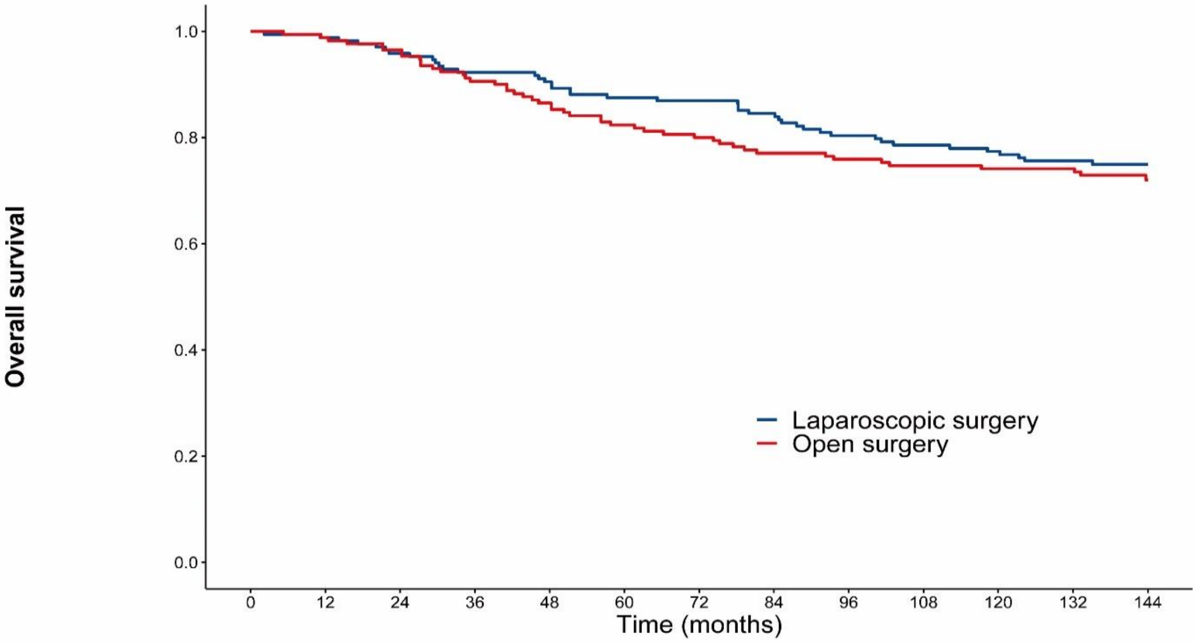

Number at risk

Time (months)	0	12	24	36	48	60	72	84	96	108	120	132	144
Laparoscopic surgery	168	166	161	155	152	147	146	142	135	132	130	122	83
Open surgery	170	168	164	154	147	140	136	131	129	127	126	121	84

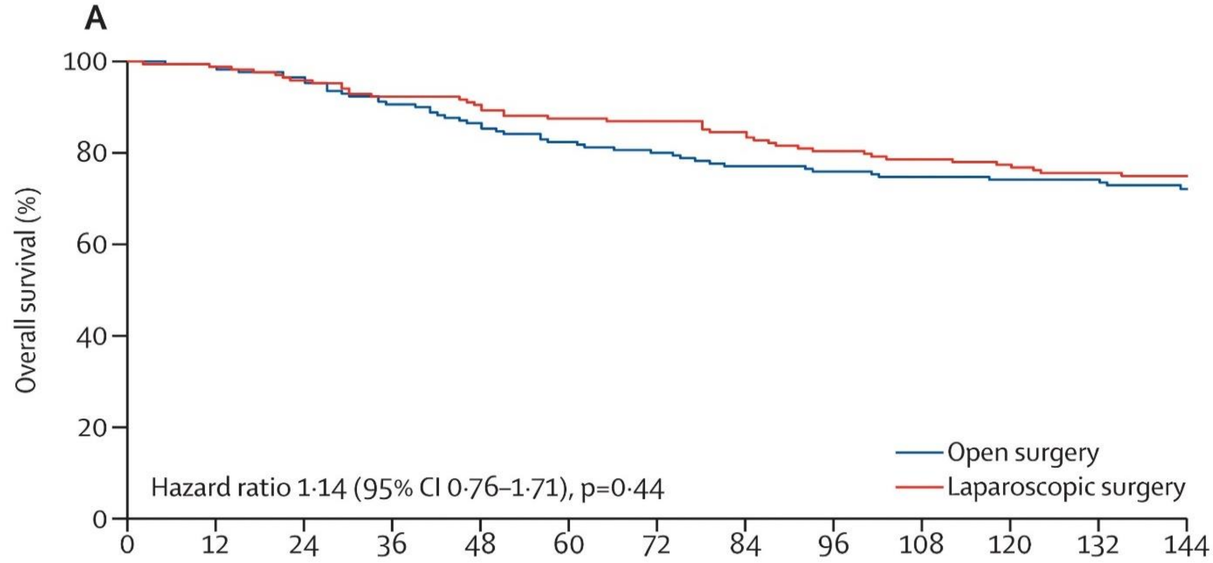

Number at risk (number censored)	0	12	24	36	48	60	72	84	96	108	120	132	144
Open surgery	170 (0)	168 (0)	164 (0)	154 (0)	147 (0)	140 (0)	136 (0)	131 (0)	129 (0)	127 (0)	126 (0)	121 (6)	84 (45)
Laparoscopic surgery	168 (0)	166 (0)	161 (0)	155 (0)	152 (0)	147 (0)	146 (0)	142 (0)	135 (0)	132 (0)	130 (0)	122 (6)	83 (48)

© 2022 Kong M et al. *JAMA Network Open*.
